# FTMT-mediated suppression of mitophagy links iron accumulation to osteoporosis

**DOI:** 10.1016/j.redox.2026.104157

**Published:** 2026-04-06

**Authors:** Ruizhi Zhang, Yike Wang, Lei Li, Junjie Li, Guangchen Feng, Yutong Hu, Gongwen Liu, Xiongyi Wang, Jiajun Zhang, Peng Wei, Houfu Lai, Keyu Zhu, Xiao Wang, Xueqin Gao, Wen Wei, Yixuan Fang, Jianrong Wang, Na Yuan, Youjia Xu

**Affiliations:** a Department of Orthopedics, The Second Affiliated Hospital of Soochow University, Suzhou, Jiangsu, China; b Research Center for Blood Engineering and Manufacturing, Cyrus Tang Medical Institute, National Research Center for Hematological Diseases, Collaborative Innovation Center of Hematology, Soochow University, Suzhou, Jiangsu, China; c Suzhou Ninth Hospital affiliated to Soochow University, Suzhou, Jiangsu, China; d Department of Orthopaedics, Suzhou TCM Hospital Affiliated to Nanjing University of Chinese Medicine, Suzhou, Jiangsu, China

**Keywords:** Iron accumulation, Mitochondrial ferritin, Bone marrow mesenchymal stem cells, Mitophagy, Osteoporosis

## Abstract

Primary osteoporosis is a major age-related disease with a significant global health burden. While iron accumulation is a known risk factor, the mechanisms linking it to bone loss remain unclear. Here, we report that impaired mitophagy in bone marrow mesenchymal stem cells (BMSCs) is a hallmark of osteoporosis and is critically exacerbated by iron accumulation. We found that iron accumulation in BMSCs inhibits mitophagy, leading to mitochondrial dysfunction, increased oxidative stress, and cellular senescence, ultimately impairing osteogenic differentiation. Importantly, targeted activation of mitophagy, either pharmacologically or genetically, restored mitochondrial health, reduced senescence, and rescued bone formation. Conversely, Pink1 deficiency in BMSCs was sufficient to induce osteoporosis. Mechanistically, we identified that the mitochondrial ferritin FTMT is upregulated under iron-loading conditions and binds to PINK1, suppressing its phosphorylation and thereby preventing mitophagy initiation. This pathway is clinically relevant, as BMSCs from osteoporotic patients with high ferritin levels showed elevated FTMT and reduced PINK1 phosphorylation. Therefore, we identify a novel pathway in which FTMT-mediated disruption of mitophagy drives iron-induced osteoporosis. Our findings highlight mitophagy activation as a therapeutic strategy to prevent and treat bone loss under iron accumulation.

## Highlights

• Clinical Correlation: BMSCs from iron accumulation-induced osteoporosis patients exhibit impaired osteogenic differentiation.
• Mechanistic Insight: Iron accumulation accelerates BMSCs senescence and suppresses osteogenesis by inhibiting mitophagy in murine models.
• PINK1 Dependency: *Pink1* deletion in BMSCs alone induced bone loss, confirming mitophagy's pivotal role.
• Molecular Pathway: Iron upregulates mitochondrial ferritin (FTMT), which binds PINK1 and inhibits its phosphorylation, disrupting mitophagy. This dual mechanism (downregulated expression and impaired function) was partially validated in human samples.
• Therapeutic Rescue: Pharmacological activation of mitophagy or *Pink1* overexpression restored BMSCs osteogenic capacity and mitigated senescence.

## Graphical abstract

Zhang et al. report that iron accumulation disrupts bone formation by upregulating FTMT, which inhibits PINK1-mediated mitophagy in bone marrow mesenchymal stem cells. This study identifies mitophagy activation as a promising therapeutic strategy for iron-related osteoporosis.

**Figure:**
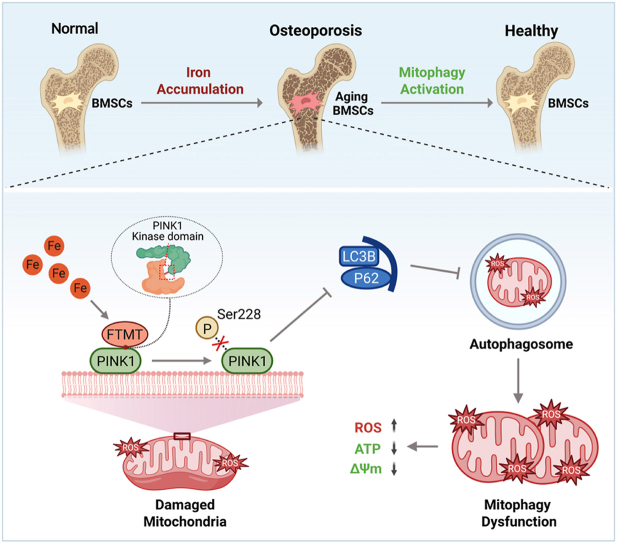

## 1 Introduction

Iron is an essential trace element, yet its dysregulation and subsequent tissue accumulation are increasingly implicated in diverse pathogenesis including osteoporosis [[1], [2], [3], [4]]. Clinical observations, such as elevated ferritin levels correlating with reduced bone mineral density in postmenopausal women and astronauts, highlight a critical, yet mechanistically unresolved, link between iron accumulation and bone loss [5,6]. Beyond these clinical findings, emerging evidence suggests that iron storage is also altered in animal models of osteoporosis. In primary osteoporosis models, particularly those driven by estrogen deficiency, dysregulated iron metabolism has been associated with bone loss and may accompany disease progression [7,8]. Similarly, in several forms of secondary osteoporosis, including unloading- or microgravity-related bone loss, iron accumulation has been implicated as a contributing factor [9,10]. The prevailing model attributes iron-induced osteoporosis to Fenton reaction-generated oxidative stress, which simultaneously suppresses osteoblast activity and promotes osteoclastogenesis [[11], [12], [13], [14]]. However, this focus on mature bone cells may overlook a more fundamental origin of the disorder within their precursors. Bone marrow mesenchymal stem cells (BMSCs) are the primary reservoir for osteoblasts, and their functional capacity, including osteogenic potential, proliferative ability, and resistance to senescence, is paramount for skeletal maintenance [[15], [16], [17]]. Growing evidence confirms that iron accumulation directly impairs BMSC function [[18], [19], [20], [21]], suggesting that the initial breakdown in bone homeostasis occurs at this stem cell level. A critical gap in knowledge is the precise molecular mechanism by which iron accumulation compromises BMSC integrity.

A promising candidate is mitochondrial quality control, particularly mitophagy. This selective autophagic process is vital for clearing damaged mitochondria, maintaining metabolic homeostasis, and counteracting cellular senescence, a key driver of age-related dysfunction [[22], [23], [24]]. The PINK1/PARKIN pathway is a principal regulator of mitophagy; PINK1 accumulation on impaired mitochondria initiates a cascade that recruits PARKIN to trigger degradation, thereby preventing the accumulation of dysfunctional organelles and reactive oxygen species (ROS) [[25], [26], [27]]. In BMSCs, robust mitophagy is essential for preserving osteogenic differentiation capacity, and its decline is directly linked to aging-related bone loss [[28], [29], [30], [31]]. We therefore hypothesized that iron accumulation disrupts bone homeostasis by impairing PINK1-mediated mitophagy, thereby inducing mitochondrial dysfunction and accelerating BMSC senescence.

In this study, we uncover a previously unidentified mechanism linking iron accumulation to osteoporosis. We found that excess iron upregulates mitochondrial ferritin (FTMT), which binds to and inhibits PINK1, a key mitophagy regulator. This suppression disrupts mitochondrial clearance, leading to oxidative stress and senescence in BMSCs. The pathway's centrality is confirmed by the osteoporotic phenotype in BMSC-specific *Pink1*-knockout mice. Therapeutically, restoring mitophagy, either pharmacologically or via *Pink1* overexpression, rescued BMSC function and bone formation. Our work defines the iron-FTMT-PINK1 axis as a core driver of bone pathology and positions mitophagy enhancement as a promising therapeutic strategy for iron-related osteoporosis.

## 2 Results

### 2.1 BMSCs from osteoporotic patients with iron accumulation exhibit impaired osteogenic function

**Fig. 1:**
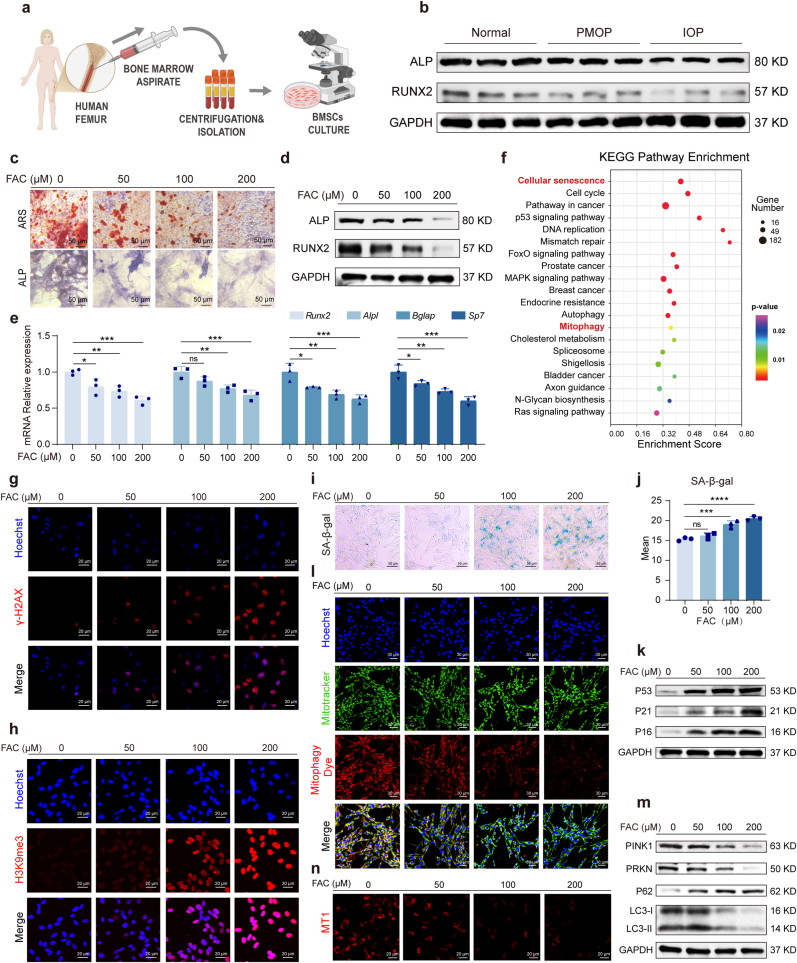
**Iron accumulation impairs mitophagy, promotes senescence, and suppresses osteogenic differentiation in BMSCs. (a)** Schematic diagram of extraction of BMSCs from human femur. **(b)** Western blot analysis of osteogenic marker proteins (RUNX2, ALP) in BMSCs from normal controls and postmenopausal osteoporosis patients and osteoporosis patients with iron accumulation. **(c)** Alizarin Red S (ARS) staining of BMSCs treated with increasing concentrations of FAC (0, 50, 100, 200 μM) for 21 days and alkaline phosphatase (ALP) staining of BMSCs treated with increasing concentrations of FAC (0, 50, 100, 200 μM) for 14 days. Scale bar: 50 μm. **(d)** Western blot analysis of osteogenic markers (RUNX2, ALP) in FAC-treated BMSCs for 5 days. **(e)** RT-qPCR analysis of osteogenic genes (*Runx2, Alpl, Bglap, Sp7*) in FAC-treated BMSCs for 72h. **(f)** KEGG pathway enrichment analysis of differentially expressed genes from RNA sequencing of control and 200 μM FAC-treated BMSCs for 72h. **(g, h)** Immunofluorescence staining of senescence markers (γ-H2AX, H3K9me3) in FAC-treated BMSCs for 72h. Scale bar: 20 μm. **(i)** Senescence-associated β-galactosidase (SA-β-gal) staining of FAC-treated BMSCs for 72h. Scale bar: 50 μm. **(j)** Flow cytometric quantification of SA-β-gal activity in FAC-treated BMSCs for 72h. **(k)** Western blot analysis of senescence-related proteins (P53, P21, P16) in FAC-treated BMSCs for 72h. **(l)** Mitophagy assessment by immunofluorescence co-staining with Mitophagy Dye (red) and MitoTracker (green) in FAC-treated BMSCs for 72h. Scale bar: 20 μm. **(m)** Western blot analysis of mitophagy/autophagy-related proteins (PINK1, PARKIN, P62, LC3) in FAC-treated BMSCs for 72h. **(n)** Mitochondrial membrane potential (MMP) detection by MT-1 staining in FAC-treated BMSCs for 72h. Scale bar: 30 μm. Data are presented as mean ± SEM; One-way ANOVA (Dunnett's multiple-comparison test); *P < 0.05, **P < 0.01, ***P < 0.001, ****P < 0.0001.

To determine the link between iron accumulation and osteogenic dysfunction in human osteoporosis, we collected bone marrow from nine female patients undergoing surgery (Fig. 1a). The demographics are summarized in Table S1. BMSCs were isolated and their identity was confirmed by flow cytometric analysis of surface markers (Fig. S1a–g). Based on ferritin levels and DXA T-scores (detected via dual-energy X-ray absorptiometry), patients were categorized into a normal bone mass group, a postmenopausal osteoporosis (PMOP) group and an iron accumulation osteoporosis (IOP) group. Western blot analysis showed that levels of key osteogenic differentiation proteins, RUNX2 and ALP, were lowest in the IOP group (Fig. 1b). These results confirm that the osteogenic function of BMSCs is compromised in osteoporotic patients with iron accumulation. Notably, this impairment is more pronounced than that observed in patients with postmenopausal osteoporosis.

### 2.2 Iron accumulation impairs osteogenic differentiation and induces senescence in BMSCs

We next investigated the direct effects of iron on BMSC function. BMSCs isolated from normal C57BL/6 mice were treated with increasing concentrations of ammonium iron citrate (FAC; 0, 50, 100, and 200 μM) for 72 h. The CCK-8 assay confirmed that these FAC concentrations did not significantly affect cell viability (Fig. S2a). To assess osteogenic differentiation, FAC-treated BMSCs were cultured in osteogenic medium. Alizarin Red S (ARS) staining revealed that FAC treatment significantly and dose-dependently reduced matrix mineralization (Fig. 1c). Consistent with this, Alkaline Phosphatase (ALP) activity, an early osteogenesis marker, was also suppressed by FAC (Fig. 1c). Quantitative analyses of ARS and ALP staining confirmed these dose-dependent impairments (Fig. S2b and c). Furthermore, both mRNA *(Runx2, Alpl, Bglap, Sp7*) and protein (RUNX2 and ALP) expression of osteogenic markers were significantly downregulated in a dose-dependent manner following FAC treatment (Fig. 1d and e).

To elucidate the mechanism behind this iron-induced dysfunction, we performed RNA sequencing on control and 200 μM FAC-treated BMSCs. We identified 4605 differentially expressed genes (2335 upregulated, 2270 downregulated) (Fig. S2d), which were visualized via a hierarchical clustering heatmap (Fig. S2e). KEGG pathway analysis highlighted significant enrichment in the cellular senescence and mitophagy pathways (Fig. 1f). Gene Ontology (GO) analysis further confirmed enrichment in cell cycle processes linked to senescence (Fig. S2f). Guided by the transcriptomic data, we directly investigated whether iron accumulation induces BMSC senescence. The expression of senescence-associated histone markers, γ-H2AX and H3K9me3, increased with rising FAC concentrations (Fig. 1g, h and S2g, h). Senescence-associated β-galactosidase (SA-β-gal) activity, a classic senescence marker, was also significantly elevated in a dose-dependent manner, as shown by both staining and flow cytometric quantification (Fig. 1i and j). Finally, we confirmed that the protein levels of key cell cycle regulators and senescence markers (P53, P21, and P16) were consistently upregulated by FAC treatment in a concentration-dependent manner (Fig. 1k). Collectively, these data demonstrate that iron accumulation not only inhibits osteogenic differentiation but also directly accelerates cellular senescence in BMSCs.

### 2.3 Iron accumulation impairs mitochondrial function and suppresses mitophagy in BMSCs

Given the link between iron, senescence, and mitochondrial health, we next investigated its impact on mitochondrial function and mitophagy in BMSCs. Mitophagy is a critical process for clearing damaged mitochondria and maintaining cellular homeostasis. Using a Mitophagy Dye that fluoresces upon lysosomal fusion with damaged mitochondria, we observed a significant, dose-dependent decrease in mitophagic activity in FAC-treated BMSCs, as shown by reduced colocalization with MitoTracker (Fig. 1l and S2i). Western blot analysis confirmed the suppression of the mitophagy pathway, evidenced by a decreased LC3-II/LC3-I ratio, downregulation of key mitophagy proteins PINK1 and PARKIN (PRKN), and accumulation of P62 (Fig. 1m). We then assessed key metrics of mitochondrial health. The mitochondrial membrane potential (MMP), measured using an MT-1-based fluorescent probe, was significantly dissipated in a dose-dependent manner upon FAC treatment (Fig. 1n and S2j). Since MMP loss is closely linked to oxidative stress, we measured reactive oxygen species (ROS) levels. Flow cytometry using DCFH-DA and Mito-SOX probes revealed that FAC treatment led to a dose-dependent increase in both intracellular and mitochondrial ROS (Fig. S2k and l). Consistent with this profound mitochondrial dysfunction, ATP production was also significantly reduced in iron-loaded BMSCs (Fig. S2m). Taken together, these findings demonstrate that iron accumulation disrupts mitochondrial function and suppresses mitophagy in BMSCs.

### 2.4 Activation of mitophagy rescues iron-induced mitochondrial dysfunction, senescence, and osteogenic impairment

**Fig. 2:**
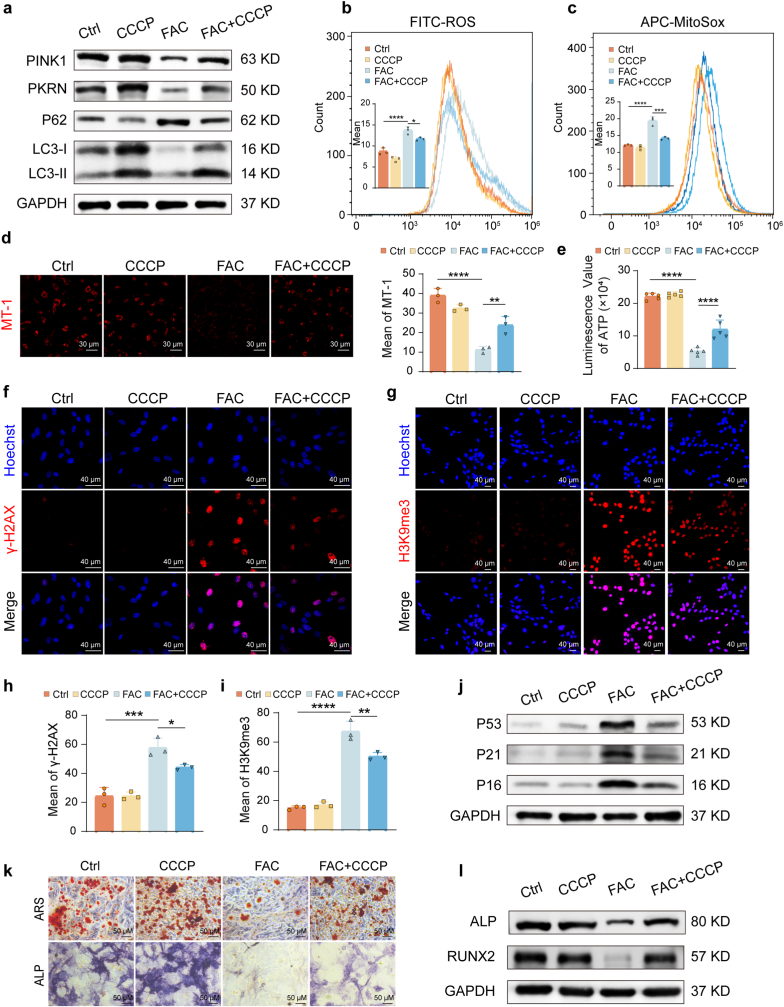
**Mitophagy activation rescues iron accumulation-induced mitochondrial dysfunction, cellular senescence, and impaired osteogenic differentiation in BMSCs.** BMSCs were isolated from normal mice and treated with 200 μM FAC with or without CCCP co-treatment for the same duration in each assay. The time points for the indicated assays were the same as those in Fig. 1. **(a)** Western blot analysis of mitophagy/autophagy-related proteins (PINK1, PARKIN, P62, LC3). **(b, c)** Flow cytometric analysis of **(b)** intracellular ROS and **(c)** mitochondrial superoxide levels. **(d)** Mitochondrial membrane potential assessment by MT-1 immunofluorescence staining. Scale bar: 30 μm. **(e)** Cellular ATP content measurement. **(f**–**i)** Immunofluorescence analysis of senescence markers (f, h) γ-H2AX and (g, i) H3K9me3. Scale bar: 40 μm. **(j)** Western blot analysis of senescence-related proteins (P53, P21, P16). **(k)** Alizarin Red S (ARS) and alkaline phosphatase (ALP) staining. Scale bar: 50 μm. **(l)** Western blot analysis of osteogenic marker proteins (RUNX2, ALP). Data are presented as mean ± SEM; One-way ANOVA (Tukey's multiple-comparison test); *P < 0.05, **P < 0.01, ***P < 0.001, ****P < 0.0001.

To determine if the detrimental effects of iron were directly attributable to deficient mitophagy, we used carbonyl cyanide 3-chlorophenylhydrazone (CCCP), an uncoupler that activates mitophagy. BMSCs were treated with FAC (200 μM) to induce iron accumulation, with or without CCCP co-treatment. We first confirmed that CCCP restored mitophagic activity, as shown by the recovery of mitophagy- and autophagy-related protein levels (LC3-II/I ratio, PINK1, PARKIN, P62) that were suppressed by FAC (Fig. 2a). This reactivation of mitophagy led to a significant improvement in mitochondrial function: CCCP attenuated the elevated levels of both intracellular and mitochondrial ROS (Fig. 2b and c), restored the mitochondrial membrane potential (Fig. 2d), and increased ATP production (Fig. 2e).

We then investigated whether this mitochondrial rescue could alleviate cellular senescence. CCCP treatment effectively reduced the expression of the senescence-associated histone markers γ-H2AX and H3K9me3 (Fig. 2f–i) and downregulated the protein levels of key senescence regulators P53, P21, and P16 in iron-loaded BMSCs (Fig. 2j). Finally, we assessed the functional recovery of osteogenic potential. ARS and ALP staining showed that CCCP treatment markedly restored the mineralization capacity and osteogenic activity that was impaired by iron accumulation (Fig. 2k), a finding supported by quantitative analysis (Fig. S3a and b). This recovery was further confirmed at the molecular level, as CCCP upregulated the expression of key osteogenic genes and proteins (Fig. 2l and S3c). These results demonstrate that pharmacological activation of mitophagy is sufficient to reverse the iron accumulation-induced mitochondrial dysfunction, cellular senescence, and impairment of osteogenic differentiation in BMSCs.

### 2.5 Mitophagy activation alleviates senescence and restores bone mass in an iron-accumulation mouse model

**Fig. 3:**
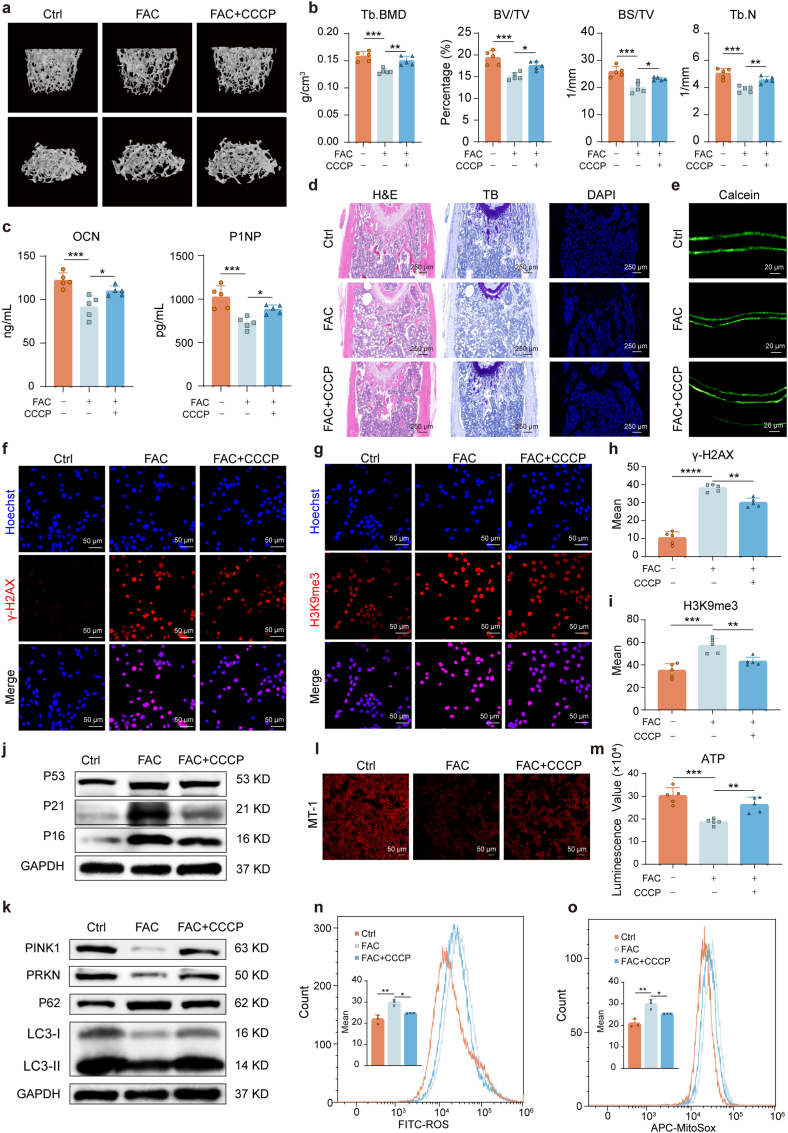
**Mitophagy activation alleviates BMSC senescence and restores bone mass in iron-accumulating mice. (a)** Representative micro-CT images of distal femoral trabecular bone. **(b)** Quantitative micro-CT analysis of trabecular bone parameters: Tb.BMD (trabecular bone mineral density), BV/TV (bone volume fraction), BS/TV (bone surface density), and Tb.N (trabecular number). **(c)** Detection of the serum OCN and P1NP levels from the mice in each group. **(d)** Histological analysis of tibial sections via H&E staining, toluidine blue staining, and DAPI immunofluorescence from the mice in each group. Scale bar: 250 μm. **(e)** Detection of the bone formation rate by calcein double labeling from the mice in each group. Scale bar: 20 μm. **(f**–**i)** Immunofluorescence analysis of senescence markers (γ-H2AX and H3K9me3) in BMSCs isolated from different treatment groups. Scale bar: 50 μm. **(j)** Western blot analysis of senescence-related proteins (P53, P21, P16) in BMSCs. **(k)** Western blot analysis of mitophagy/autophagy-related proteins (PINK1, PARKIN, P62, LC3) in BMSCs. **(l)** Mitochondrial membrane potential assessment by MT-1 immunofluorescence staining in BMSCs. Scale bar: 50 μm. **(m)** Cellular ATP content measurement in BMSCs. **(n**–**o)** Flow cytometric analysis of **(n)** intracellular ROS and **(o)** mitochondrial superoxide levels in BMSCs. Data are presented as mean ± SEM; One-way ANOVA (Tukey's multiple-comparison test); *P < 0.05, **P < 0.01, ***P < 0.001, ****P < 0.0001.

To validate our *in vitro* findings, we investigated whether mitophagy activation could mitigate iron-induced osteoporosis *in vivo*. We established a mouse model of iron-accumulation-induced osteoporosis and treated the mice with the mitophagy activator CCCP as an intervention, based on previous studies [[32], [33], [34], [35]]. Successful iron accumulation was confirmed by Prussian blue staining of the liver (Fig. S4a). After two months, micro-CT analysis of femurs revealed that iron accumulation caused significant bone loss and microarchitectural deterioration, characterized by reduced trabecular bone mineral density (Tb. BMD), bone volume fraction (BV/TV), bone surface-to-volume ratio (BS/TV), and trabecular number (Tb.N) (Fig. 3a and b). Crucially, CCCP treatment effectively mitigated this bone loss, preserving bone mass and microstructure. This restoration of bone integrity was further supported by histological analyses (H&E, toluidine blue, and DAPI staining), which showed that CCCP reversed the iron-induced thinning and fragmentation of trabeculae (Fig. 3d). Consistently, serum levels of osteogenic markers (OCN and P1NP) were suppressed by iron accumulation but restored by CCCP treatment (Fig. 3c), and calcein double labeling confirmed improved bone formation rates (Fig. 3e). The functional mechanical strength of the bones, assessed by a three-point bending test, was also compromised by iron accumulation (reduced maximum load and stiffness) but was significantly recovered with CCCP intervention (Fig. S4b and c).

We next isolated BMSCs from these mice to examine the cellular mechanisms. BMSCs from iron-accumulating mice showed a significant increase in the senescence markers γ-H2AX and H3K9me3, which was effectively reversed by CCCP treatment (Fig. 3f–i). Western blot analysis further confirmed that CCCP delayed BMSC senescence *in vivo* (Fig. 3j). Further, Western blot analysis confirmed that CCCP enhanced autophagy and mitophagy pathways in these cells *in vivo* (Fig. 3k). CCCP intervention restored mitochondrial function in the isolated BMSCs, as evidenced by the recovery of mitochondrial membrane potential and ATP production (Fig. 3l, m and S4d), alongside a reduction in both intracellular and mitochondrial ROS (Fig. 3n and o). Detection of osteogenic marker expression at both the mRNA (*Runx2, Alpl, Bglap, Sp7*) and protein (RUNX2, ALP) levels showed that CCCP restored the osteogenic capacity of BMSCs from iron-accumulating mice (Fig. S4e and f). Collectively, these results demonstrate that pharmacological activation of mitophagy *in vivo* rescues iron accumulation-induced osteoporosis by improving mitochondrial function, reducing BMSC senescence, restoring BMSC osteogenic capacity and promoting bone formation.

### 2.6 Genetic deletion of *Pink1* in BMSCs is sufficient to cause osteoporosis

**Fig. 4:**
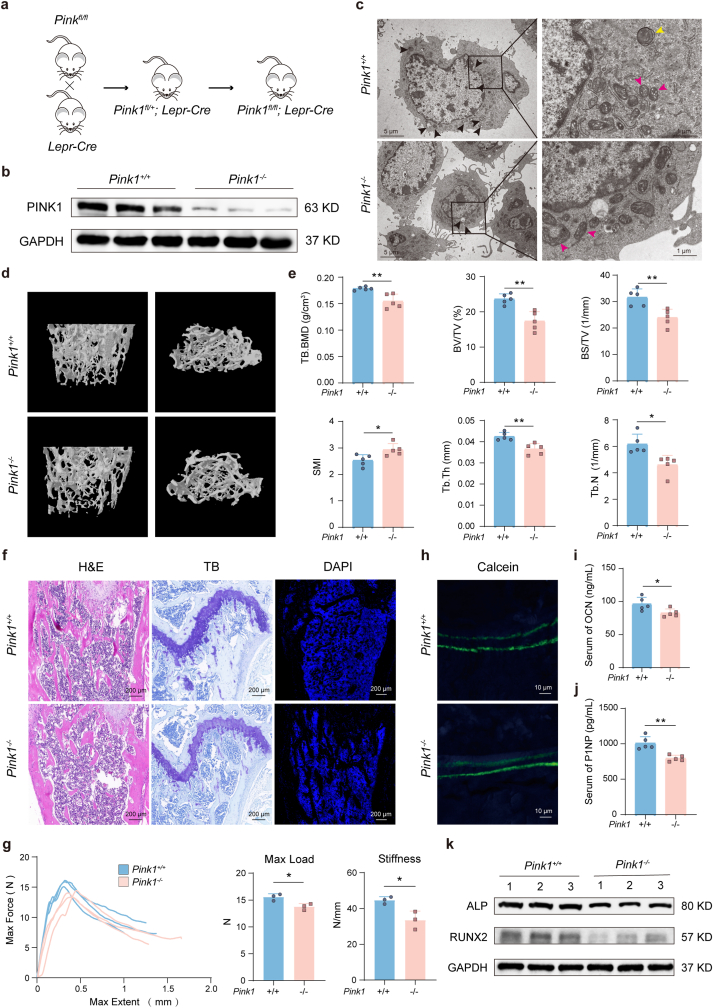
**BMSC-specific PINK1 deficiency induces bone loss independent of iron accumulation. (a)** Schematic of the breeding strategy to generate BMSC-specific Pink1 conditional knockout mice (*Pink1*^*fl/fl*^; Lepr-Cre). **(b)** Western blot validation of *Pink1* knockout efficiency in isolated BMSCs. **(c)** Transmission electron microscopy images of BMSCs showing mitochondrial ultrastructure and autophagic vesicles. Black triangles: autophagosomes; pink triangles: mitochondria; yellow triangles: mitochondria within autophagosomes (mitophagosomes). **(d)** Representative micro-CT reconstruction images of distal femoral trabecular bone. **(e)** Quantitative micro-CT analysis of trabecular bone parameters: Tb.BMD (trabecular bone mineral density), BV/TV (bone volume fraction), BS/TV (bone surface density), SMI (Structure Model Index), Tb.Th (trabecular thickness) and Tb.N (Trabecular Number). **(f)** Histological analysis of tibial sections by H&E staining, toluidine blue staining, and DAPI immunofluorescence. Scale bar: 200 μm. **(g)** Biomechanical properties assessed by three-point bending test, showing maximum load and stiffness. **(h)** Detection of the bone formation rate by calcein double labeling from the mice. Scale bar: 20 μm. **(i**–**j)** Detection of the serum OCN and P1NP levels from the mice. **(k)** Western blot analysis of osteogenic marker proteins (RUNX2, ALP) in BMSCs. Data are presented as mean ± SEM; Unpaired 2-tailed Student's *t-*test; *P < 0.05, **P < 0.01.

Our data strongly implicate impaired PINK1-mediated mitophagy in iron-induced osteoporosis. To establish whether a primary defect in mitophagy is causative, we genetically ablated *Pink1* specifically in BMSCs by crossing *Pink1*^*fl/fl*^ mice with Lepr-Cre mice (Fig. 4a and S5a, b). BMSCs isolated from the resulting *Pink1*^*fl/fl*^; Lepr-Cre mice confirmed successful knockout at the protein level (Fig. 4b). Transmission electron microscopy (TEM) revealed severe mitochondrial abnormalities in *Pink1*-deficient BMSCs, including swollen, misshapen mitochondria and a notable reduction in lysosomes, indicating a fundamental failure in mitochondrial quality control (Fig. 4c). Strikingly, this BMSC-specific *Pink1* deletion was sufficient to cause a low bone mass phenotype. Micro-CT analysis showed that *Pink1*^*fl/fl*^; Lepr-Cre mice exhibited significantly reduced bone mass and disrupted microarchitecture compared to *Pink1*^*fl/fl*^ controls, with lower Tb. BMD, BV/TV, BS/TV, trabecular thickness (Tb.Th), Tb.N and higher structure model index (SMI) (Fig. 4d and e). Histological staining (H&E, toluidine blue) and DAPI immunofluorescence of femoral sections confirmed the degradation of bone microstructure in the knockout mice (Fig. 4f). The bones of *Pink1*^*fl/fl*^; Lepr-Cre mice were also mechanically weaker, exhibiting lower maximum load and stiffness in three-point bending tests (Fig. 4g). This osteoporotic phenotype was driven by impaired bone formation, as shown by a decreased bone formation rate via calcein double labeling (Fig. 4h) and reduced serum levels of OCN and P1NP (Fig. 4i and j). Accordingly, the expression of osteogenic genes and proteins was significantly downregulated in *Pink1*-null BMSCs (Fig. 4k and S5c). Importantly, there were no significant differences in bone resorption parameters (serum β-CTX levels, TRAP staining) between the genotypes (Fig. S5d and e), indicating the phenotype is primarily due to a defect in bone formation. Together, these results demonstrate that the loss of *Pink1* in BMSCs is sufficient to recapitulate the osteoporotic phenotype, independent of iron accumulation, establishing PINK1 as a critical regulator of bone mass through its role in maintaining BMSC function.

### 2.7 Pink1 overexpression rescues iron-induced BMSC dysfunction

**Fig. 5:**
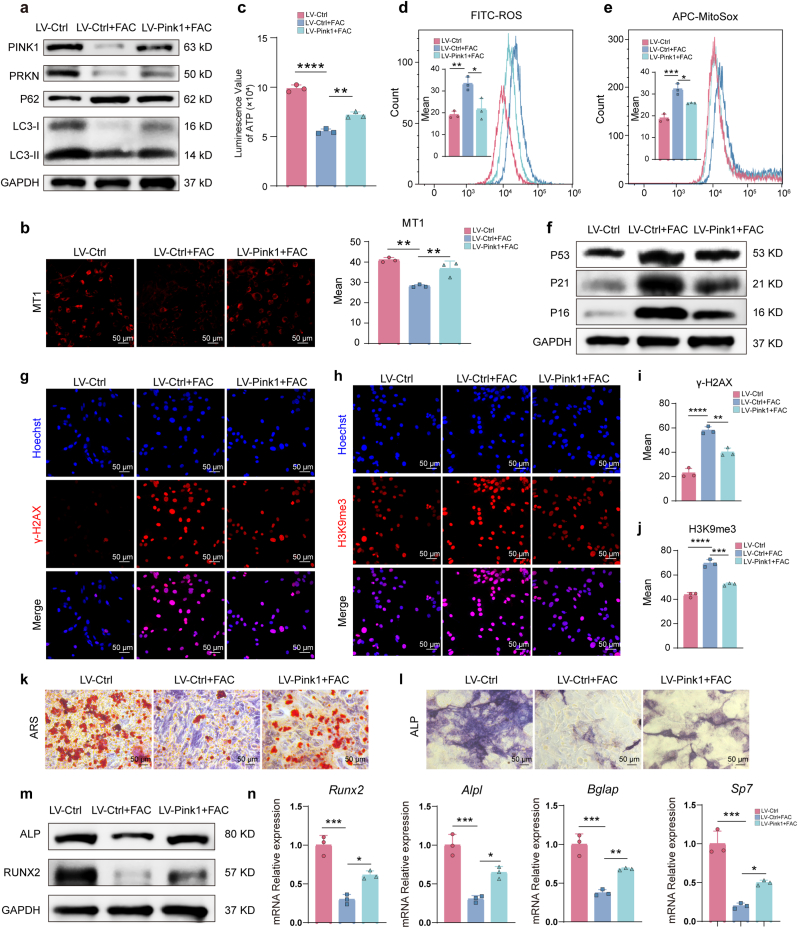
**PINK1 overexpression rescues iron accumulation-induced mitochondrial dysfunction, senescence, and osteogenic impairment in BMSCs.** The time points for the indicated assays were the same as those in Fig. 1. **(a)** Western blot analysis of mitophagy/autophagy-related proteins (PINK1, PARKIN, P62, LC3) in BMSCs transduced with control or PINK1-overexpressing lentivirus followed by FAC treatment. **(b)** Mitochondrial membrane potential assessment by MT-1 immunofluorescence staining. Scale bar: 50 μm. **(c)** Cellular ATP content measurement. **(d, e)** Flow cytometric analysis of (d) intracellular ROS and (e) mitochondrial superoxide levels. **(f)** Western blot analysis of senescence-related proteins (P53, P21, P16). **(g**–**j)** Immunofluorescence analysis of senescence markers (γ-H2AX and H3K9me3). Scale bar: 50 μm. **(k, l)** Alizarin Red S (ARS) staining and Alkaline phosphatase (ALP) staining. Scale bar: 50 μm. **(m)** Western blot analysis of osteogenic marker proteins (RUNX2, ALP). **(n)** RT-qPCR analysis of osteogenic genes (*Runx2, Alpl, Bglap, Sp7*). Data are presented as mean ± SEM; One-way ANOVA (Tukey's multiple-comparison test); *P < 0.05, **P < 0.01, ***P < 0.001, ****P < 0.0001.

Given that *Pink1* deletion causes osteoporosis, we asked whether restoring *Pink1* could reverse the damage from iron accumulation. We transduced BMSCs with a lentiviral vector to overexpress *Pink1* prior to FAC treatment. High transduction efficiency was confirmed by GFP immunofluorescence (Fig. S6a), and successful *Pink1* overexpression was verified by Western blot and RT-qPCR (Fig. 5a and S6b). Notably, *Pink1* overexpression ameliorated the iron-induced suppression of mitophagy. Functionally, it restored the mitochondrial membrane potential (Fig. 5b) and ATP production (Fig. 5c) that were impaired by FAC. It also significantly reduced the elevated levels of both intracellular and mitochondrial ROS (Fig. 5d and e). We then assessed whether this mitochondrial rescue alleviated cellular senescence. *Pink1* overexpression effectively reduced the expression of senescence-related proteins (Fig. 5f) and attenuated the fluorescence intensity of the senescence markers γ-H2AX and H3K9me3 in iron-loaded BMSCs (Fig. 5g–j). Crucially, this reversal of senescence translated into a recovery of osteogenic function. *Pink1* overexpression restored the mineralization capacity and osteogenic activity that were suppressed by FAC, as shown by ARS and ALP staining (Fig. 5k, l and S6c, d). This was further confirmed by the upregulation of osteogenic genes and proteins (Fig. 5m and n). These results demonstrate that targeted *Pink1* overexpression is sufficient to counteract iron accumulation by restoring mitophagy, improving mitochondrial health, reducing senescence, and rescuing the osteogenic potential of BMSCs.

### 2.8 FTMT suppresses mitophagy by directly inhibiting PINK1 phosphorylation

**Fig. 6:**
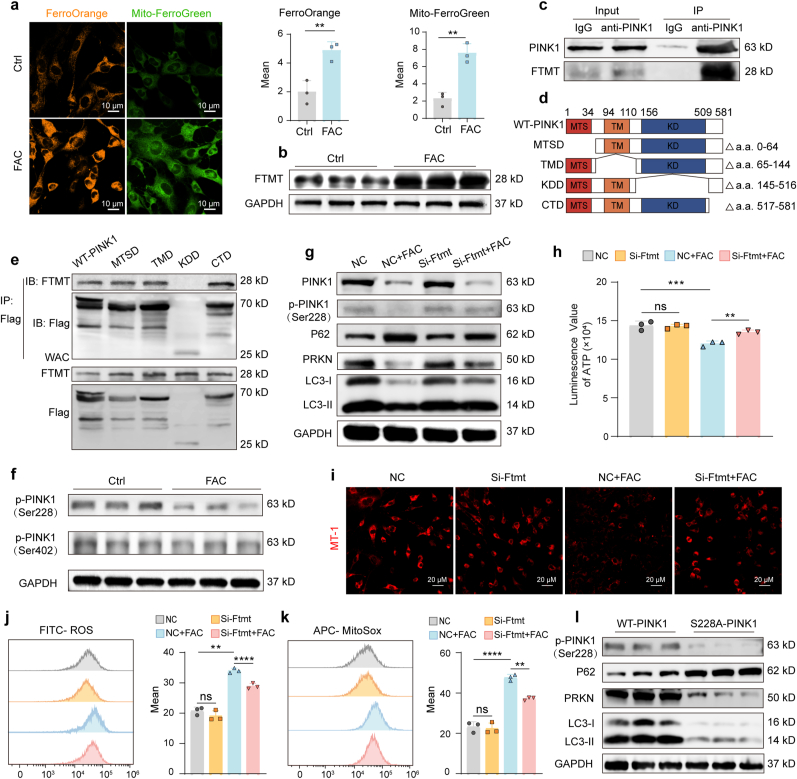
**FTMT upregulation during iron accumulation impairs mitophagy by inhibiting PINK1 phosphorylation. (a)** Immunofluorescence detection of intracellular and mitochondrial iron levels in BMSCs. Scale bar: 10 μm. **(b)** Western blot analysis of FTMT expression in BMSCs with or without FAC treatment. **(c)** Co-immunoprecipitation analysis of PINK1-FTMT interaction in BMSCs treated with FAC. **(d)** Schematic diagram of full-length and domain-deletion mutants of PINK1 (MTS: mitochondrial targeting sequence; TM: transmembrane domain; KD: kinase domain). **(e)** Co-immunoprecipitation using anti-Flag antibody in BMSCs transfected with WT-PINK1 or PINK1 deletion mutants and treated with FAC, followed by FTMT detection. **(f)** Western blot analysis of PINK1 phosphorylation at Ser228 and Ser402 in BMSCs. **(g)** Western blot analysis of mitophagy/autophagy-related proteins (PINK1, p-PINK1(Ser228), PARKIN, P62, LC3) in control and FTMT-knockdown BMSCs under iron accumulation. **(h)** Cellular ATP content measurement. **(i)** Mitochondrial membrane potential assessment by MT-1 immunofluorescence staining. Scale bar: 20 μm. **(j, k)** Flow cytometric analysis of **(j)** intracellular ROS and **(k)** mitochondrial superoxide levels. **(l)** Western blot analysis of mitophagy/autophagy-related proteins (p-PINK1(Ser228), PARKIN, P62, LC3) in BMSCs expressing PINK1 with S228A point mutation. Data are presented as mean ± SEM; Unpaired 2-tailed Student's *t*-test (a), One-way ANOVA (Tukey's multiple-comparison test) (h, j and k); **P < 0.01, ***P < 0.001, ****P < 0.0001.

We next sought the mechanism by which iron accumulation inhibits mitophagy. We first confirmed that FAC treatment elevated both intracellular and mitochondrial iron levels (Fig. 6a). This was accompanied by a significant upregulation of mitochondrial ferritin (FTMT), a key protein for mitochondrial iron storage (Fig. 6b). To elucidate how FTMT impairs mitophagy, we investigated its interaction with PINK1. Co-immunoprecipitation (Co-IP) assays revealed a direct interaction between FTMT and PINK1 specifically under iron-accumulation conditions (Fig. 6c). To map the interaction domain, we generated a series of PINK1 domain mutants (Fig. 6d). Co-IP assays with these constructs identified the kinase domain (KD) of PINK1 as the specific binding site for FTMT (Fig. 6e). Since this domain is critical for PINK1 activation, we examined its autophosphorylation. Iron accumulation markedly decreased phosphorylation at the Ser228 residue within the kinase domain, while phosphorylation at Ser402 was unaffected (Fig. 6f). To establish a causal link, we knocked down *Ftmt* using siRNA, confirming high knockdown efficiency (Fig. S7a and b). In iron-loaded BMSCs, *Ftmt* knockdown did not alter total PINK1 levels but robustly increased phosphorylation at Ser228. This recovery of PINK1 activity was accompanied by restored expression of mitophagy-related proteins (P62, PARKIN, LC3), indicating a reactivation of the pathway (Fig. 6g). Concomitantly, this activation of the mitophagy pathway restored ATP production and mitochondrial membrane potential while reducing both intracellular and mitochondrial ROS levels (Fig. 6h–k and S7c). Furthermore, introducing a point mutation at the PINK1 Ser228 site was sufficient to shift the profile of mitophagy proteins toward an inhibited state, mimicking the effect of iron accumulation (Fig. 6l). These data establish a precise molecular mechanism: iron accumulation upregulates FTMT, which then binds to the kinase domain of PINK1 and specifically inhibits its phosphorylation at Ser228, thereby blocking the initiation of mitophagy.

### 2.9 The FTMT-PINK1 mitophagy axis is impaired in patient-derived BMSCs

**Fig. 7:**
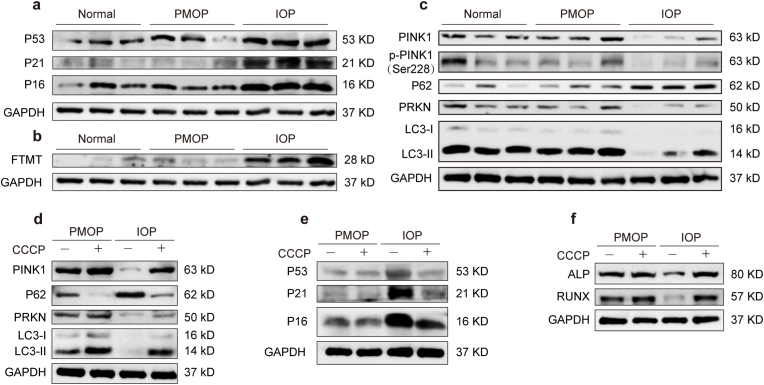
**Impaired mitophagy in BMSCs from osteoporosis patients with iron accumulation. (a)** Western blot analysis of senescence-related proteins (P53, P21, P16) in BMSCs from normal controls, postmenopausal osteoporosis patients and osteoporosis patients with iron accumulation. **(b)** Western blot analysis of mitochondrial ferritin (FTMT) expression levels in BMSCs. **(c)** Western blot analysis of mitophagy/autophagy-related proteins PINK1, p-PINK1(Ser228), PARKIN, P62, and LC3 in BMSCs. **(d)** Western blot analysis of mitophagy/autophagy-related proteins PINK1, PARKIN, P62, and LC3 in BMSCs of PMOP and IOP group with or without CCCP intervention. **(e)**Western blot analysis of senescence-related proteins (P53, P21, P16) in BMSCs of PMOP and IOP group with or without CCCP intervention. **(f)** Western blot analysis of osteogenic marker proteins (RUNX2, ALP) in BMSCs of PMOP and IOP group with or without CCCP intervention.

To validate the clinical relevance of our findings, we analyzed BMSCs from our cohort of osteoporosis patients. Western blot analysis confirmed that BMSCs from the iron accumulation group exhibited elevated levels of senescence-related proteins compared to the normal group (Fig. 7a). Critically, the key components of our proposed pathway were consistently dysregulated in these patient samples. BMSCs from the iron accumulation group showed significant upregulation of FTMT (Fig. 7b). Concurrently, they displayed a marked downregulation in the mitophagy pathway, characterized by reduced levels of total PINK1, phospho-PINK1 (Ser228), PARKIN, and LC3-II, alongside an accumulation of P62 (Fig. 7c). The expression levels of the above proteins showed no significant differences between the normal and PMOP groups. The concordance of these human data with our *in vitro* and *in vivo* results strongly supports the pathophysiological significance of the FTMT-PINK1-mitophagy axis in human osteoporosis associated with iron accumulation. Furthermore, BMSCs from the PMOP and IOP groups were treated with the mitophagy agonist. Western blot analysis confirmed that mitophagy levels were markedly increased in BMSCs from both groups after CCCP intervention (Fig. 7d). In the iron accumulation group, CCCP intervention led to decreased expression of senescence-associated proteins and increased expression of osteogenesis-related proteins, whereas no significant changes were observed in the PMOP group (Fig. 7e and f). These data further support the potential of targeting mitophagy as a therapeutic strategy for iron accumulation osteoporosis.

## 3 Discussion

Primary osteoporosis, an age-related disorder characterized by reduced bone mass and microstructural deterioration, poses a significant fracture risk for the elderly [36,37]. The pathogenesis of this condition hinges on disrupted bone remodeling, a process in which BMSCs play a pivotal role [[38], [39], [40]]. In this study, we delineate a novel molecular pathway through which iron accumulation accelerates osteoporosis. We demonstrate that iron accumulation triggers the upregulation of FTMT, which directly binds to and inhibits the phosphorylation of PINK1 at Ser228, thereby suppressing the initiation of mitophagy. This mitophagy defect leads to the accumulation of dysfunctional mitochondria, driving BMSC senescence and impairing osteogenic differentiation. The critical role of PINK1 is underscored by our finding that BMSC-specific *Pink1* knockout mice exhibit profound bone loss. Importantly, we provide two independent therapeutic proofs-of-concept: both pharmacological activation of mitophagy and genetic *Pink1* overexpression successfully rescue the iron-induced phenotypes. Our work thus defines a pathogenic iron-FTMT-PINK1-mitophagy axis in osteoporosis and nominates the enhancement of mitophagy as a viable therapeutic strategy.

Iron accumulation induces BMSC senescence through mitochondrial dysfunction. The detrimental role of iron in bone metabolism, primarily attributed to Fenton reaction-derived ROS that promote osteoclast activity and suppress osteoblast function, is well-established [8,[41], [42], [43], [44]]. Our findings significantly extend this paradigm by demonstrating that the damaging effects of iron extend upstream to osteoblast progenitors, the BMSCs. We provide comprehensive evidence that iron accumulation dose-dependently induces a senescent phenotype in BMSCs, marked by elevated β-galactosidase activity, increased P53/P21/P16 expression, and persistent DNA damage. This aligns with the recognized role of mitochondrial dysfunction as a key driver of cellular senescence [45,46]. Our data confirm that iron accumulation severely compromises mitochondrial integrity in BMSCs, as evidenced by elevated mitochondrial ROS, diminished ATP production, and a reduced mitochondrial membrane potential (ΔΨm). Crucially, we identified a concurrent and marked suppression of mitophagy, a key quality control mechanism. This defect, characterized by reduced PINK1/PARKIN signaling and P62 accumulation, provides a mechanistic explanation for the accrual of damaged mitochondria and the subsequent progression of BMSC senescence, a phenomenon previously observed in aged BMSCs [47].

PINK1 is a central regulator of bone homeostasis and a key target of iron toxicity. The PINK1/PARKIN pathway is a well-characterized regulator of mitophagy, where PINK1 stabilization on damaged mitochondria initiates a cascade that recruits PARKIN to orchestrate mitochondrial clearance [[48], [49], [50]]. The importance of this pathway in bone is increasingly appreciated; for instance, its enhancement via LRRc17 silencing has been shown to rejuvenate aged BMSCs [51]. Furthermore, Guo et al. reported that advanced glycation end products accelerate BMSC aging by inhibiting PINK1/PARKIN-mediated mitophagy [52]. Modulating PINK1/Parkin-mediated mitophagy by targeting ANT1 mitigated the progression of trauma-induced tendon heterotopic ossification [53]. Furthermore, Yang et al. demonstrated that DDIT3 deficiency suppresses mtROS production by enhancing PINK1/Parkin-dependent mitophagy, thereby protecting against osteoarthritis [54]. Our study solidifies the indispensability of PINK1 in bone by demonstrating that its specific deletion in BMSCs is sufficient to cause a low bone mass and fragility phenotype in mice. More importantly, we uncover a novel mechanism of PINK1 dysregulation in the context of iron accumulation. We found that iron accumulation does not merely downregulate PINK1 expression but, more precisely, inhibits its critical kinase activity.

FTMT serves as a molecular bridge linking iron accumulation to PINK1 inhibition. The upregulation of FTMT is a recognized compensatory response to mitochondrial iron accumulation [55]. However, our study reveals a previously unknown detrimental consequence of this adaptation. We discovered that FTMT physically interacts with the kinase domain of PINK1, and this interaction selectively impedes PINK1 phosphorylation at the Ser228 residue, a site critical for its full activation and downstream signaling [[56], [57], [58]]. This site-specific inhibition, which leaves Ser402 phosphorylation and total PINK1 levels unaffected, represents a sophisticated form of pathway regulation. The functional consequence of this interaction is profound, as silencing *Ftmt* restored PINK1-Ser228 phosphorylation and reactivated the mitophagy cascade, while a Ser228 point mutation mimicked the inhibitory effects of iron accumulation. This FTMT-PINK1 interaction provides a direct molecular link explaining how iron disrupts mitochondrial quality control, offering a more precise mechanism than general oxidative stress.

The translational significance of our proposed axis is strongly supported by data from human BMSCs isolated from osteoporotic patients with iron accumulation. These cells consistently recapitulated our experimental findings, exhibiting elevated FTMT, reduced PINK1-Ser228 phosphorylation, impaired mitophagy, and increased senescence markers. From a therapeutic perspective, our successful rescue of the iron-accumulation phenotype through both a pharmacological agent (CCCP) and genetic (*Pink1* overexpression) approaches provides compelling pre-clinical evidence that targeting the PINK1-mitophagy axis holds promise. Furthermore, the elucidation of the FTMT-PINK1 interaction interface opens the door for future structure-based drug design. Inhibiting this specific protein-protein interaction could represent a novel strategy to restore mitophagy and combat iron-induced osteoporosis, potentially with greater specificity than general antioxidants or iron chelators.

In summary, our study moves beyond establishing a correlation between iron and bone loss to delineating a detailed causal pathway. We identify FTMT as a critical molecular sensor of iron accumulation that directly impairs the core mitophagy machinery by inhibiting PINK1. This not only advances understanding of osteoporotic pathogenesis but also redefines FTMT from an iron buffer to an active regulator of cell signaling, with implications for other age-related and iron-associated disorders.

### 3.1 Limitation of the study

A limitation of this study is that our mechanistic analyses were performed primarily in BMSCs. While BMSCs are currently the mainstream and most widely used model for investigating osteogenic differentiation and its molecular regulation, they may not fully reflect the heterogeneity of stem/progenitor cells across distinct skeletal microenvironments [59]. Future studies are therefore warranted to examine whether iron accumulation exerts similar effects on other skeletal stem/progenitor populations, including periosteal stem cells and skeletal stem cells. Osteoporosis is a heterogeneous disorder, and the contribution of iron dysregulation likely varies across disease subtypes. While elevated ferritin levels and iron accumulation have been linked to bone loss in several clinical and experimental settings, these alterations should not be assumed to be uniform across all forms of osteoporosis. The magnitude, timing, and pathogenic significance of ferritin-associated changes may differ depending on the underlying etiology, including primary osteoporosis related to aging or estrogen deficiency, as well as secondary osteoporosis resulting from metabolic, inflammatory, unloading, or drug-induced insults [60]. Therefore, our findings are best interpreted as defining a mechanistic iron-responsive pathway in BMSCs, rather than implying that ferritin or iron-storage abnormalities are identical across all osteoporosis subtypes.

## 4 Methods

### 4.1 Experimental design

Commercially acquired BMSCs (Cyagen Biosciences) were used for *in vitro* experiments. To induce iron accumulation, cells were treated with various concentrations (0, 50, 100, or 200 μM) of ferric ammonium citrate (FAC; Honeywell, 1185-57-5). For RNA sequencing, untreated and 200 μM FAC-treated BMSCs were selected. For mitophagy activation studies, four treatment groups were established: Control; CCCP (10 μM, MCE, 100941) for 72 h; FAC (200 μM) for 72 h; and FAC + CCCP (co-treatment for 72 h). For *Pink1* overexpression, three groups were used: LV-Control (transfected with empty lentiviral vector); LV-Control + FAC (transfected with empty vector, then treated with 200 μM FAC for 72 h); and LV-Pink1+FAC (transfected with *Pink1*-overexpressing lentivirus, then treated with 200 μM FAC for 72 h).

### 4.2 Isolation and culture of BMSCs

For *in vitro* studies, BMSCs were cultured in complete α-MEM (Gibco, C12571500BT) supplemented with 10% fetal bovine serum (Procel, 164210-50) and 1% penicillin/streptomycin (NCM Biotech, C100C5). For *in vivo* studies, BMSCs were isolated from mouse femurs and tibias by flushing the bone marrow and culturing cells in complete α-MEM with 20% fetal bovine serum. Cultures were maintained at 37 °C in a 5% CO_2_ environment, with medium changes every other day.

### 4.3 Identification of human BMSCs

Human BMSCs were identified by flow cytometry. Cells were stained with fluorochrome-conjugated antibodies against CD105, CD29, CD73 (positive markers), and CD11b, CD45, CD34 (negative markers). A population was defined as BMSCs if ≥ 90% of cells were positive for the positive markers and ≤5% were positive for the negative markers.

### 4.4 Cell viability assay

BMSCs were seeded in 96-well plates (5000 cells/well) and treated with FAC for 72 h. Cell viability was assessed using a CCK-8 kit (Dojindo, CK04), and absorbance was measured at 450 nm with a microplate reader (SpectraMax M5, USA).

### 4.5 RNA sequencing (RNA-seq)

Total RNA was extracted with TRIzol (Beyotime, R0016). RNA-seq libraries were prepared and sequenced by BGI, China on a BGISEQ-500 platform. Differential gene expression analysis, along with KEGG and GO enrichment analyses, was performed as previously described [61].

### 4.6 Osteogenic differentiation and staining

BMSCs were induced with osteogenic medium containing 50 μg/ml ascorbate-2 (Sigma, PHR1008), 10 mM β-glycerol phosphate (Abmole, M3837), and 0.1 μM dexamethasone (Abmole, M2176). ALP staining (Beyotime, C3206) was performed on day 14, and Alizarin Red S (ARS) staining (Cyagen Biosciences, ALIR-10001) was performed on day 21. RNA and protein were harvested on days 3 and 5 of differentiation, respectively, for RT-qPCR and Western blot analysis.

### 4.7 Western blot analysis

Cells were lysed, and proteins were separated by SDS-PAGE, transferred to PVDF membranes (Millipore, IPVH00010), and probed with primary antibodies against: RUNX-2 (Abcam, ab236639), ALP (Affinity, DF6225), P53 (Affinity, AF0879), P21 (Affinity, DF6423), P16 (Abcam, ab51243), PINK1 (HUABIO, ER1706-27), PARKIN (HUABIO, ET1702-60), P62 (Abcam, ab109012), LC3 (NOVUS, NB100-2220), FTMT (Abmart, PC20086S), Phospho-PINK1[Ser228] (Cell Signaling, 89010T), Phospho-PINK1[Ser402] (Absin, abs148820), and GAPDH (Affinity, AF7021). Blots were developed with HRP-conjugated secondary antibodies (Cell Signaling, 7074S) and an ECL kit (Fude Biological Technology, FD800).

### 4.8 RT-qPCR analysis

RNA was reverse-transcribed into cDNA (Vazyme, R323-01), and RT-qPCR was performed using SYBR Green MasterMix (Vazyme, Q412-02) on an ABI7900 system. Gene expression was normalized to Gapdh. The primers used were as follows: Alpl, 5′-CCAGAAAGACACCTTGACTGTGG-3′ and 5′-TCTTGTCCGTGTCGCTCACCAT-3′; Bglap, 5′-AGGAGGGCAATAAGGTAGTG-3′ and 5′-TGTAGGCGGTCTTCAAGC-3′; Runx2, 5′-CCTGAACTCTGCACCAAGTCCT-3′ and 5′-TCATCTGGCTCAGATAGGAGGG-3′; Sp7, 5′-GGCAAGGCTTCGCATCTG-3′ and 5′-CTCAAGTGGTCGCTTCTGG-3′; Pink1, 5′-CGACAACATCCTTGTGGAGTGG-3′ and 5′-CATTGCCACCACGCTCTACACT-3′; Ftmt, 5′-GCTTCCTCTCAGGACTCCACTA-3′ and 5′-TGGACAGGTACACGTAGGATGC-3′; Gapdh, 5′-TCAACGGCACAGTCAAGG-3′ and 5′-ACTCCACGACATACTCAGC-3′.

### 4.9 β-Galactosidase staining

After treatment for 72 h, the BMSCs were fixed with 4% formaldehyde and stained with a Senescence β-Galactosidase Staining Kit (BeyoTime, C0602) following the protocol published by the manufacturer.

β-Galactosidase quantification.

After treatment for 72 h, the BMSCs were collected for quantification of β-galactosidase activity. After two washes in HBSS, the BMSCs were incubated for the designated times with the Cellular Senescence Detection Kit-SPiDER-βGal (Dojindo, SG03) prior to measurement by flow cytometry. Logarithmic amplification was used to measure SPiDER-βGal fluorescence.

### 4.10 Immunofluorescence

Cells were fixed, permeabilized with 0.1% Triton X-100 (Sigma‒Aldrich, V900502), and incubated with anti-γ-H2AX (Abcam, ab289) or anti-H3K9me3 (Abcam, ab176916) antibodies, followed by fluorophore-conjugated secondary antibodies (Affinity, S0006). Nuclei were counterstained with Hoechst 33342 (Invitrogen, 62249). Images were captured by confocal microscopy and analyzed with ImageJ.

### 4.11 Mitochondrial membrane potential (MMP) assessment

MMP was measured using the MT-1 MitoMP Detection Kit (Dojindo, MT13). Fluorescence was visualized by confocal microscopy and quantified with ImageJ.

### 4.12 Mitophagy analysis

Mitophagy in BMSCs was analyzed via a mitophagy detection kit (Dojindo, MD01). Then, 100 nmol/L Mitophagy Dye (containing 100 nmol/L MitoTracker Green Probe [Dojindo, MT10]) was added to each group, and the cells were incubated for 30 min. After experimentation, the cells were washed twice with Hank's solution. Finally, the nuclei were stained with Hoechst 33342 (Invitrogen, 62249) for 10 min at room temperature without exposure to light. The fluorescence was measured via confocal microscopy, and the level of fluorescence was determined via ImageJ.

### 4.13 Measurement of adenosine triphosphate (ATP) content

Briefly, the BMSCs were cultured in 96-well culture plates, and the plates were incubated at room temperature for approximately 30 min. One hundred microliters of CellTiter-Glo® Reagent (Promega, G7570) was added to each well, and the contents were mixed for 2 min on a shaker to induce cell lysis. The plate was incubated at room temperature for 10 min to stabilize the luminescent signal, and then the luminescence was recorded via a multifunctional microplate reader (SpectraMax M5, USA).

### 4.14 Analysis of the ROS level

DCFH-DA (Invitrogen, C6827) was used for intracellular ROS detection, and MitoSOX Red (Invitrogen, M36009) was used to determine mitochondrial superoxide. BMSCs were collected and incubated with 10 μmol/L DCFH-DA or 5 μmol/L MitoSOX Red working solution at 37 °C for 30 min. The solution was mixed by inversion every 5 min to ensure full contact of the probe with the cells. Flow cytometry analysis was used to measure fluorescence.

### 4.15 Mice

Mice lacking mitophagy in BMSCs were generated by crossing mice hemizygous for the Lepr-Cre transgene with mice heterozygous for a *Pink1*-flox allele (purchased from Gempharmatech) to generate heterozygous *Pink1*-flox offspring with and without the Lepr-Cre allele. These offspring were crossed to generate wild-type mice (*Pink1*^*+/+*^), hemizygous for the Lepr-Cre allele, and homozygous for the *Pink1*-flox allele, with Pink1 deletion in BMSCs (*Pink1*^*−/−*^). The female mice were euthanized at 6 months of age for subsequent experiments. The genotype of the isolated BMSCs was verified via western blotting. For the iron accumulation model, 8-week-old C57BL/6 female mice were intraperitoneally injected with 0.1 g kg^−1^ weekly FAC for 8 weeks. CCCP was injected at a dose of 0.3 mg kg^−1^ weekly when FAC was injected. The mice were bred and housed in the specific-pathogen-free animal facilities of Soochow University. All animal experiments were approved by the institutional animal care and use committee of Soochow University.

### 4.16 Prussian blue staining

Liver paraffin sections were stained to detect iron content via the Prussian Blue Iron Stain Kit (Solarbio, G1422) according to the manufacturer's instructions.

### 4.17 Bone micro-CT image

Femurs were scanned and analyzed for trabecular bone via a micro-CT system (SkyScan, Bruker, Kontich, Belgium). The acquisition parameters were as follows: X-ray voltage = 50 kV, X-ray current = 800 μA, filter = 0.5 mm aluminum, rotation step = 0.7, and image pixel size = 9.3 μm. After scanning, images were reconstructed via NRecon software (Bruker, Kontich, Belgium). The parameters of the trabecular bone were determined via CTAn software (Bruker, Kontich, Belgium), and 3D image reconstruction was performed via CTvOX software (Bruker, Kontich, Belgium). For trabecular bone parameters, the volume of interest (VOI = 100 slices) was selected with reference to the distal growth plate. The trabecular bone regions started approximately 0.7 mm from the growth plate and extended toward the proximal end of the femur. The cancellous bone parameters of the femoral metaphysis included the trabecular BMD (Tb.BMD), bone volume-to-total tissue volume ratio (BV/TV), bone surface-to-total tissue volume ratio (BS/TV), structure model index (SMI), trabecular thickness (Tb.Th) and trabecular number (Tb.N).

### 4.18 Bone histology

Tissues were fixed in 10% formalin overnight, decalcified with 20% EDTA for 2 weeks, and processed for paraffin sectioning. Tibiae were collected and fixed overnight at 4 °C and embedded undecalcified in methyl methacrylate. Five-micron-thick sections were processed for hematoxylin and eosin (H&E) staining, toluidine blue (TB) staining, DAPI immunofluorescence staining and TRAP staining.

### 4.19 Biomechanical properties

The biomechanical properties of the femora were measured via three-point bending and compression testing. Femora were collected and stored at −20 °C. Femora were tested via three-point bending with the posterior surface on the lower supports (5 mm apart), and the load was applied to the anterior surface centered between the lower supports, as previously described in detail (Akhter, Cullen, Gong,& Recker, 2001). Biomechanical structural strength variables, including the ultimate load and stiffness, were measured.

### 4.20 Transmission electron microscopy

The BMSCs were fixed in 2.5% electron microscopy grade glutaraldehyde in 0.1 M sodium cacodylate buffer. Transmission electron microscopy images were obtained via Servicebio technology.

### 4.21 Calcein labeling

The mice were intraperitoneally injected with 10 mg/kg calcein in a 1% saline solution for 10 or 3 days before they were sacrificed. The femurs were fixed overnight in 4% paraformaldehyde, dehydrated in 30% sucrose for 2 days, and sectioned for calcein labeling.

### 4.22 ELISA analysis

Mouse serum was obtained by collecting venous blood from the retro-orbital venous plexus after the mice were anesthetized with sodium pentobarbital. The collected blood was then centrifuged at 3000 rpm for 10 min at 4 °C, and the supernatant was collected for further use. The levels of β-CTX (Enzyme-linked Biotechnology, China), P1NP (Enzyme-linked Biotechnology, China), and OCN (Enzyme-linked Biotechnology, China) in the serum were measured via ELISA kits according to the manufacturer's instructions.

### 4.23 Small interfering RNA (siRNA) transfection

To silence *Pink1* and *Ftmt* expression, BMSCs were transfected with scrambled siRNA (30 nM) or target gene siRNA (30 nM) (GenePharma, China) for 48 h via Hiperfect Transfection Reagent (Qiagen; cat. no. 301705) according to the manufacturer's instructions.

### 4.24 Lentivirus transfection

*Pink1* was overexpressed via the transfection of *Pink1*-overexpressing lentivirus (OBiO Technology, China). BMSCs were transfected with *Pink1*-overexpressing lentivirus or vehicle lentivirus at a confluence of 30–50%. After 12 h, the medium was changed, and the cells were further incubated for 3 days until they reached 90–95% confluence. The passaged cells were used in subsequent experiments. Efficacies of transfection were determined via Western blot analysis.

### 4.25 Plasmid transfection

All plasmids, including wild-type and mutant variants of PINK1 and point mutation of PINK1 at Ser228 site, were procured from XIEBHC Biotechnology. BMSCs were transfected with transfection reagent, mixed with cDNA as per the manufacturer's instructions.

### 4.26 Co-immunoprecipitation

The BMSCs were lysed with IP lysis buffer (Beyotime Biotechnology, P0013) for total protein, after which the cell supernatants were pretreated with protein A/G agarose beads (Beyotime Biotechnology, P2012) for 1–2 h at 4 °C. Then, the cell supernatants were incubated with 0.5–1 μg of anti-Pink1 overnight at 4 °C. Next, 45–50 μl of protein A/G agarose was added for 4 h. The beads were washed more than three times with IP lysis buffer, and the bound proteins were boiled in loading buffer for further analysis.

### 4.27 Detection of iron

The fluorescent probes FerroOrange (Dojindo, F374) and Mito-FerroGreen (Dojindo, M489) were used to detect the free Fe^2+^ content in the cytoplasm and mitochondria. After 1.0 × 10^5^ cells were incubated with 1 μM FerroOrange working solution in a 12-well plate for 30 min, they were visualized directly with a fluorescence microscope.

### 4.28 Human sample

A total of 9 human femur bone marrow samples were obtained from patients who underwent surgery. Bone marrow was collected during hip replacement surgery or knee replacement surgery. Patients who had hip fractures caused by trauma or falls, and osteoarthritis were included in our study (inclusion criteria). Subjects with secondary osteoporosis, anti-osteoporosis drug treatment, a disrupted hematopoietic system, malignancy, diabetes, or other severe diseases in the previous 5 years were excluded from our study (exclusion criteria). Osteoporosis was defined on the basis of bone mineral density (BMD), per WHO standards (BMD T value <−2.5). Areal BMD in the hip was measured via dual-energy X-ray absorptiometry (DXA). hBMSCs were immediately isolated and purified after enzymatic digestion. The cells were then filtered through a nylon membrane and counted before being seeded into 10-cm dishes with complete α-MEM supplemented with 20% fetal bovine serum and 1% penicillin/streptomycin. The medium was changed every other day. The cultures were maintained at 37 °C with 5% CO2.

### 4.29 Statistical analysis

For animal experiments, sample size was estimated based on the primary bone phenotype outcomes. For *in vitro* experiments, sample size was determined based on the principal quantitative readouts of each assay. Statistical analysis was conducted using GraphPad software (version 9.01). Statistical methods included t-tests or one-way analysis of variance (ANOVA). A *P* value < 0.05 was considered statistically significant. All data are presented as mean ± standard error of the mean (SEM). All representative experiments were repeated at least three times.

### 4.30 Study approval

The patients were from the Second Affiliated Hospital of Soochow University, and all clinical procedures were approved by the Ethics Committees of the Second Affiliated Hospital of Soochow University (Approval no. JD-LK-2020-027-0). We also obtained informed consent from the participants. The animal experiments were reviewed and approved by the Institutional Committee on Animal Welfare Protection and Ethics of Soochow University.

## Funding

This work was supported by the 10.13039/501100001809National Natural Science Foundation of China (82372455, 82470165); Jiangsu Provincial Medical Key Laboratory Cultivation Unit (JSDW202254); The special project of “Technological innovation” project of 10.13039/501100014883China National Nuclear Corporation Medical Industry (ZHYLZD2023001); Tang Scholar Program.

## CRediT authorship contribution statement

**Ruizhi Zhang:** Formal analysis, Investigation, Writing – original draft. **Yike Wang:** Investigation, Writing – original draft. **Lei Li:** Formal analysis, Methodology. **Junjie Li:** Formal analysis, Methodology. **Guangchen Feng:** Formal analysis, Validation. **Yutong Hu:** Formal analysis, Methodology. **Gongwen Liu:** Formal analysis, Validation. **Xiongyi Wang:** Formal analysis, Validation. **Jiajun Zhang:** Formal analysis, Validation. **Peng Wei:** Formal analysis, Validation. **Houfu Lai:** Formal analysis, Validation. **Keyu Zhu:** Formal analysis, Validation. **Xiao Wang:** Formal analysis, Validation. **Xueqin Gao:** Formal analysis, Validation. **Wen Wei:** Formal analysis, Validation. **Yixuan Fang:** Formal analysis, Writing – review & editing. **Jianrong Wang:** Conceptualization, Supervision, Writing – review & editing. **Na Yuan:** Conceptualization, Funding acquisition, Supervision, Writing – review & editing. **Youjia Xu:** Conceptualization, Funding acquisition, Project administration, Supervision, Writing – review & editing.

## Declaration of competing interest

The authors have declared no conflict of interest.

## Data Availability

Data will be made available on request.

## References

[1] Galy B., Conrad M., Muckenthaler M. (2024). Mechanisms controlling cellular and systemic iron homeostasis. Nat. Rev. Mol. Cell Biol..

[2] Grote Beverborg N. (2019). Differences in clinical profile and outcomes of low iron storage vs defective iron utilization in patients with heart failure: results from the DEFINE-HF and BIOSTAT-CHF studies. JAMA Cardiol..

[3] Gao H., Jin Z., Bandyopadhyay G. (2022). Aberrant iron distribution via hepatocyte-stellate cell axis drives liver lipogenesis and fibrosis. Cell Metab..

[4] Zhang H. (2024). The influence of iron on bone metabolism disorders. Osteoporos. Int..

[5] Kim B.J. (2012). Iron overload accelerates bone loss in healthy postmenopausal women and middle-aged men: a 3-year retrospective longitudinal study. J. Bone Miner. Res..

[6] Zwart S.R. (2013). Iron status and its relations with oxidative damage and bone loss during long-duration space flight on the International Space Station. Am. J. Clin. Nutr..

[7] Xiao W., Beibei F., Guangsi S. (2015). Iron overload increases osteoclastogenesis and aggravates the effects of ovariectomy on bone mass. J. Endocrinol..

[8] Wang X., Chen B., Sun J. (2018). Iron-induced oxidative stress stimulates osteoclast differentiation via NF-κB signaling pathway in mouse model. Metabolism.

[9] Yang J., Zhang G., Dong D., Shang P. (2018). Effects of iron overload and oxidative damage on the musculoskeletal System in the space environment: data from spaceflights and ground-based simulation models. Int. J. Mol. Sci..

[10] Zhang J., Zhao H., Yao G., Qiao P., Li L., Wu S. (2021). Therapeutic potential of iron chelators on osteoporosis and their cellular mechanisms. Biomed. Pharmacother..

[11] Ru Q. (2023). Fighting age-related orthopedic diseases: focusing on ferroptosis. Bone Res..

[12] Dong Y. (2024). A clinical-stage Nrf2 activator suppresses osteoclast differentiation via the iron-ornithine axis. Cell Metab..

[13] Xia Y. (2023). REPIN1 regulates iron metabolism and osteoblast apoptosis in osteoporosis. Cell Death Dis..

[14] Das B.K. (2022). Transferrin receptor 1-mediated iron uptake regulates bone mass in mice via osteoclast mitochondria and cytoskeleton. eLife.

[15] Zhu S. (2024). Cell signaling and transcriptional regulation of osteoblast lineage commitment, differentiation, bone formation, and homeostasis. Cell Discov..

[16] Wang D. (2024). Cell membrane vesicles derived from hBMSCs and hUVECs enhance bone regeneration. Bone Res..

[17] Xu Y. (2024). USP26 combats age-related declines in self-renewal and multipotent differentiation of BMSC by maintaining mitochondrial homeostasis. Adv. Sci. (Weinh.).

[18] Yang F. (2017). Melatonin protects bone marrow mesenchymal stem cells against iron overload-induced aberrant differentiation and senescence. J. Pineal Res..

[19] Yu Z.Y. (2018). Heme oxygenase-1 protects bone marrow mesenchymal stem cells from iron overload through decreasing reactive oxygen species and promoting IL-10 generation. Exp. Cell Res..

[20] Jing Z., Li Y., Zhang H. (2023). Tobacco toxins induce osteoporosis through ferroptosis. Redox Biol..

[21] Jiang Z. (2024). Ferroptosis in osteocytes as a target for protection against Postmenopausal osteoporosis. Adv. Sci. (Weinh.).

[22] Ma Y. (2018). Autophagy controls mesenchymal stem cell properties and senescence during bone aging. Aging Cell.

[23] Picca A., Faitg J., Auwerx J., Ferrucci L., D'Amico D. (2023). Mitophagy in human health, ageing and disease. Nat. Metab..

[24] Gustafsson Å.B., Dorn G.W. (2019). Evolving and expanding the roles of mitophagy as a homeostatic and pathogenic process. Physiol. Rev..

[25] Narendra D.P., Youle R.J. (2024). The role of PINK1-Parkin in mitochondrial quality control. Nat. Cell Biol..

[26] Ashrafi G., Schwarz T.L. (2013). The pathways of mitophagy for quality control and clearance of mitochondria. Cell Death Differ..

[27] Clague M.J., Urbé S. (2025). Diverse routes to mitophagy governed by ubiquitylation and mitochondrial import. Trends Cell Biol..

[28] Xiang K. (2024). Tobacco toxins trigger bone marrow mesenchymal stem cells aging by inhibiting mitophagy. Ecotoxicol. Environ. Saf..

[29] Chen H. (2024). SCUBE3 promotes osteogenic differentiation and mitophagy in human bone marrow mesenchymal stem cells through the BMP2/TGF-β signaling pathway. FASEB J..

[30] Wu X. (2024). Hypoxia-induced mitochondrial fission regulates the fate of bone marrow mesenchymal stem cells by maintaining HIF1α stabilization. Free Radic. Biol. Med..

[31] Wang J. (2025). Alda-1 mediates cell senescence and counteracts bone loss in weightlessness through regulating mitophagy. Life Sci..

[32] Huang Y. (2025). BMP9 alleviates iron accumulation-induced osteoporosis via the USP10/FOXO1/GPX4 axis. J. Adv. Res..

[33] Wang Y. (2025). COPB1 deficiency triggers osteoporosis with elevated iron stores by inducing osteoblast ferroptosis. J. Orthop. Transl..

[34] Song X. (2025). Inhibition of mitophagy via the EIF2S1-ATF4-PRKN pathway contributes to viral encephalitis. J. Adv. Res..

[35] Tang M. (2025). A positive feedback loop between SMAD3 and PINK1 in regulation of mitophagy. Cell Discov..

[36] Yu B., Wang C.Y. (2016). Osteoporosis: the result of an 'aged' bone microenvironment. Trends Mol. Med..

[37] Zhang R. (2025). Biological age acceleration predicts osteoporosis and reduced longevity in a large prospective cohort. Bone.

[38] Zhang Y. (2021). Neuronal induction of bone-fat imbalance through osteocyte Neuropeptide Y. Adv. Sci. (Weinh.).

[39] Ma S. (2023). Skeletal muscle-derived extracellular vesicles transport glycolytic enzymes to mediate muscle-to-bone crosstalk. Cell Metab..

[40] Yu S. (2024). Time of exercise differentially impacts bone growth in mice. Nat. Metab..

[41] Cui Z. (2025). Targeting Irgm1 to combat osteoporosis: suppressing ROS and restoring bone remodeling. Cell Death Dis..

[42] Riegger J. (2023). Oxidative stress as a key modulator of cell fate decision in osteoarthritis and osteoporosis: a narrative review. Cell. Mol. Biol. Lett..

[43] Guo S. (2016). Iron homeostasis: transport, metabolism, and regulation. Curr. Opin. Clin. Nutr. Metab. Care.

[44] Kim H. (2023). Transferrin receptor-mediated iron uptake promotes colon tumorigenesis. Adv Sci.

[45] Ravindran R., Gustafsson Å.B. (2025). Mitochondrial quality control in cardiomyocytes: safeguarding the heart against disease and ageing. Nat. Rev. Cardiol..

[46] Li X., Luo X., Cao X. (2025). Sirtuins in Parkinson's disease: molecular mechanisms and pathophysiological roles. Ageing Res. Rev..

[47] Miwa S. (2022). Mitochondrial dysfunction in cell senescence and aging. J. Clin. Investig..

[48] Zhang F. (2020). P53 and Parkin co-regulate mitophagy in bone marrow mesenchymal stem cells to promote the repair of early steroid-induced osteonecrosis of the femoral head. Cell Death Dis..

[49] Geisler S. (2010). PINK1/Parkin-mediated mitophagy is dependent on VDAC1 and p62/SQSTM1. Nat. Cell Biol..

[50] Matsuda N. (2010). PINK1 stabilized by mitochondrial depolarization recruits Parkin to damaged mitochondria and activates latent Parkin for mitophagy. J. Cell Biol..

[51] Liu F., Yuan Y., Bai L. (2021). LRRc17 controls BMSC senescence via mitophagy and inhibits the therapeutic effect of BMSCs on ovariectomy-induced bone loss. Redox Biol..

[52] Guo Y. (2021). Sirt3-mediated mitophagy regulates AGEs-induced BMSCs senescence and senile osteoporosis. Redox Biol..

[53] Li G., Li T., Deng Y. (2025). Targeting ANT1 to regulate PINK1/Parkin-mediated mitophagy is an effective treatment of trauma-induced tendon heterotopic ossification. J. Orthop. Transl..

[54] Yang C., Ke J., Xu Q. (2026). Mitochondrial DNA released from pyroptotic synovial macrophages via DDIT3-mediated mitophagy aggravates osteoarthritis progression. J. Orthop. Transl..

[55] Wang P. (2022). Mitochondrial ferritin alleviates apoptosis by enhancing mitochondrial bioenergetics and stimulating glucose metabolism in cerebral ischemia reperfusion. Redox Biol..

[56] Okatsu K. (2012). PINK1 autophosphorylation upon membrane potential dissipation is essential for Parkin recruitment to damaged mitochondria. Nat. Commun..

[57] Rasool S. (2022). Mechanism of PINK1 activation by autophosphorylation and insights into assembly on the TOM complex. Mol Cell.

[58] Raimi O.G. (2024). Mechanism of human PINK1 activation at the TOM complex in a reconstituted system. Sci. Adv..

[59] Li X., Liang T., Dai B. (2024). Excess glucocorticoids inhibit murine bone turnover via modulating the immunometabolism of the skeletal microenvironment. J. Clin. Investig..

[60] Li X., Xu J., Dai B., Wang X., Guo Q., Qin L. (2020). Targeting autophagy in osteoporosis: from pathophysiology to potential therapy. Ageing Res. Rev..

[61] Trapnell C. (2012). Differential gene and transcript expression analysis of RNA-seq experiments with TopHat and cufflinks. Nat. Protoc..

