# Supplementary material for: FTMT-mediated suppression of mitophagy links iron accumulation to osteoporosis
Source: Redox Biol. 2026 Apr 6;93:104157. doi: 10.1016/j.redox.2026.104157 (PMC13100270; doi:10.1016/j.redox.2026.104157)
Supplement: Multimedia component 1 [file mmc1.pdf]

Supplemental Data

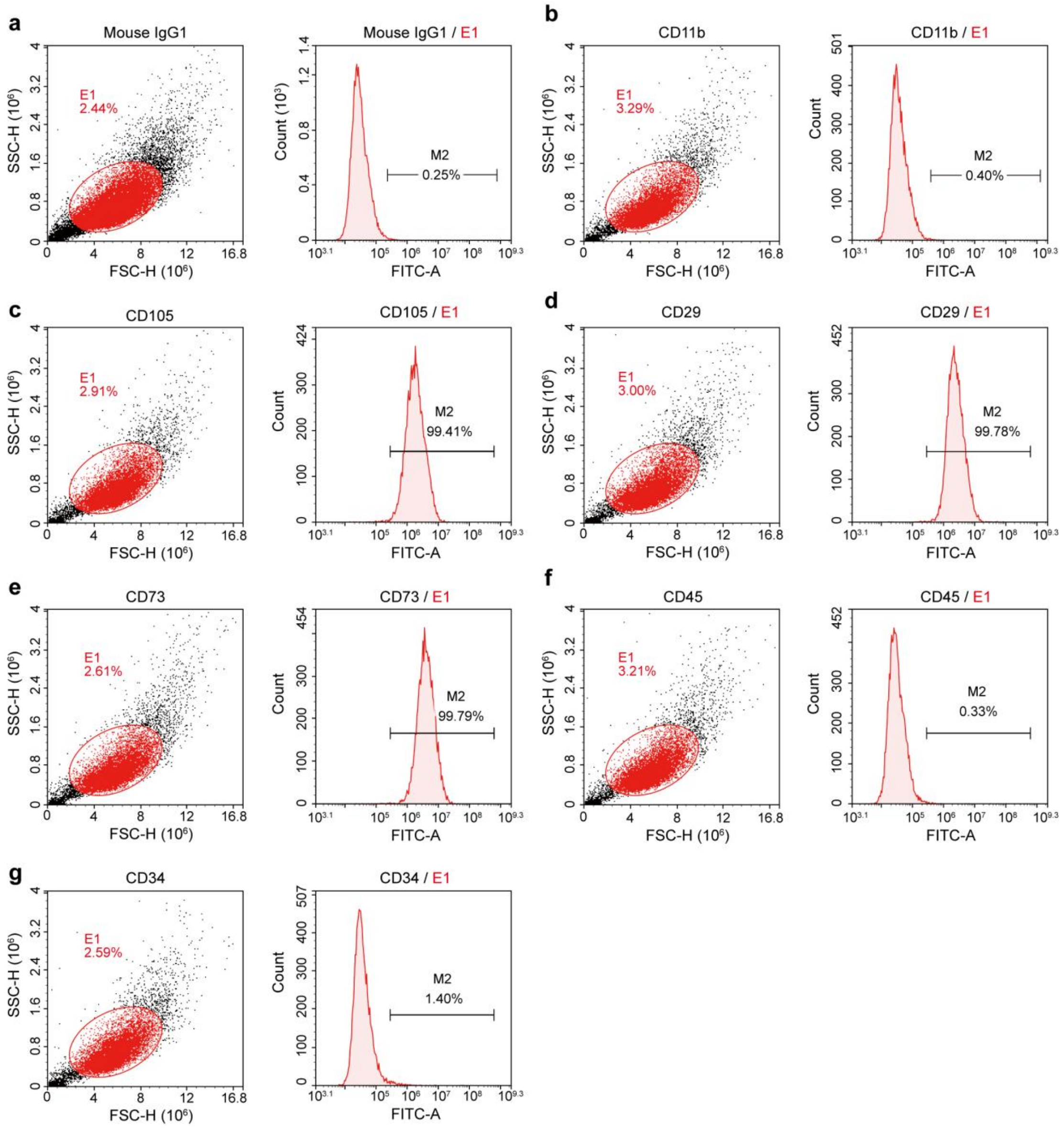

Figure S1. Identification of human BMSCs by flow cytometry. Representative flow cytometry plots showing the expression of various cell surface markers on BMSCs. Each panel includes a dot plot (left) and a corresponding histogram (right) to quantify marker expression. **(a)** Mouse IgG1 as an isotype control, showing 2.44% positive cells in region E1. **(b)** CD11b expression, with 3.29% of cells in region E1 and 0.4% of cells in region M2. **(c)** CD105 expression, with 2.91% of cells in region E1 and 99.41% of cells in region M2. **(d)** CD29 expression, with 3.00% of cells in

region E1 and 99.78% of cells in region M2. **(e)** CD73 expression, with 2.61% of cells in region E1 and 99.79% of cells in region M2. **(f)** CD45 expression, with 3.21% of cells in region E1 and 0.33% of cells in region M2. **(g)** CD34 expression, with 2.59% of cells in region E1 and 1.40% of cells in region M2.

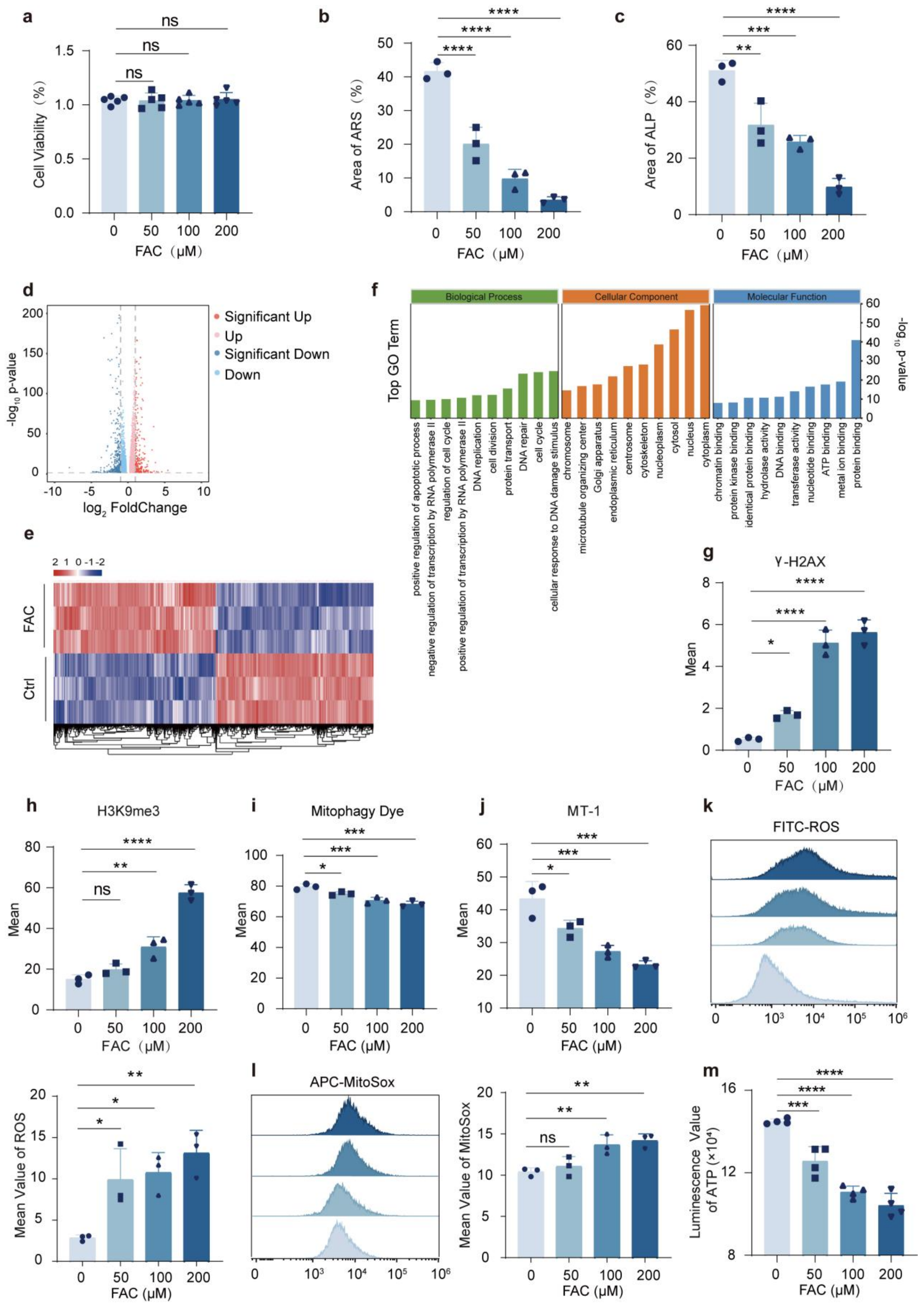

Figure S2. Iron accumulation inhibits mitophagy, accelerates senescence, and suppresses the osteogenic differentiation of BMSCs. The time points for the indicated assays were the same as those in Figure 1. **(a)** Viability of BMSCs treated

with various concentrations of FAC. **(b)** Quantitative analysis of the ARS staining area in BMSCs treated with various concentrations of FAC. **(c)** Quantitative analysis of the ALP staining area in BMSCs treated with various concentrations of FAC. **(d)** Volcano plot depicting differentially expressed genes (DEGs) between 200 μM FAC-treated BMSCs and untreated controls. **(e)** Heatmap visualization of DEGs between BMSCs treated with 200 μM FAC and control BMSCs. **(f)** GO pathway enrichment analysis of DEGs between BMSCs treated with 200 μM FAC and control BMSCs. **(g, h)** Quantitative analysis of immunofluorescence staining (γ-H2AX and H3K9me3). **(i)** Quantitative analysis of immunofluorescence staining (Mitophagy Dye). **(j)** Quantitative analysis of immunofluorescence staining (MT-1). **(k, l)** Flow cytometry detection of intracellular and mitochondrial oxidative stress levels in BMSCs treated with various concentrations of FAC. **(m)** Detection of ATP content in BMSCs treated with various concentrations of FAC. Data are expressed as mean ± SEM; One-way ANOVA (Dunnett's multiple-comparison test); *$P < 0.05$; **$P < 0.01$; ***$P < 0.001$; ****$P < 0.0001$.

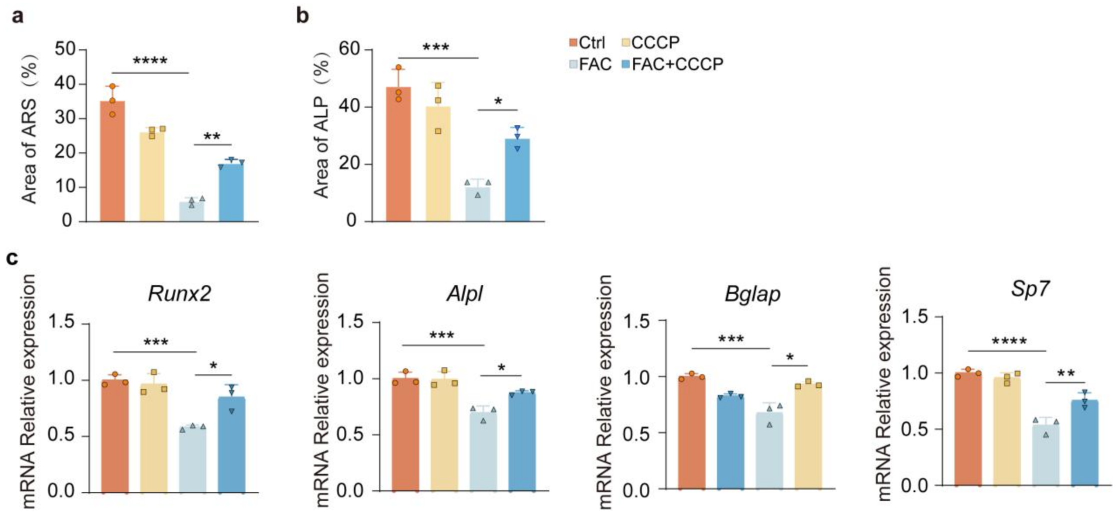

Figure S3. Mitophagy activation rescues iron accumulation-induced impaired osteogenic differentiation in BMSCs. BMSCs were isolated from normal mice and treated with 200 μM FAC with or without CCCP co-treatment for the same duration in each assay. The time points for the indicated assays were the same as those in Figure 1. **(a)** Quantitative analysis of the ALP-stained area in the BMSCs from each group (Ctrl group, CCCP group, FAC group, FAC+CCCP group). **(b)** Quantitative analysis of the ARS-stained area in the BMSCs from each group. **(c)** RT-qPCR quantification of osteogenesis marker genes (*Runx2, Alpl, Bglap, and Sp7*) in BMSCs from each group. Data are expressed as mean ± SEM; One-way ANOVA (Tukey's multiple-comparison test); *$P < 0.05$; **$P < 0.01$; ***$P < 0.001$; ****$P < 0.0001$.

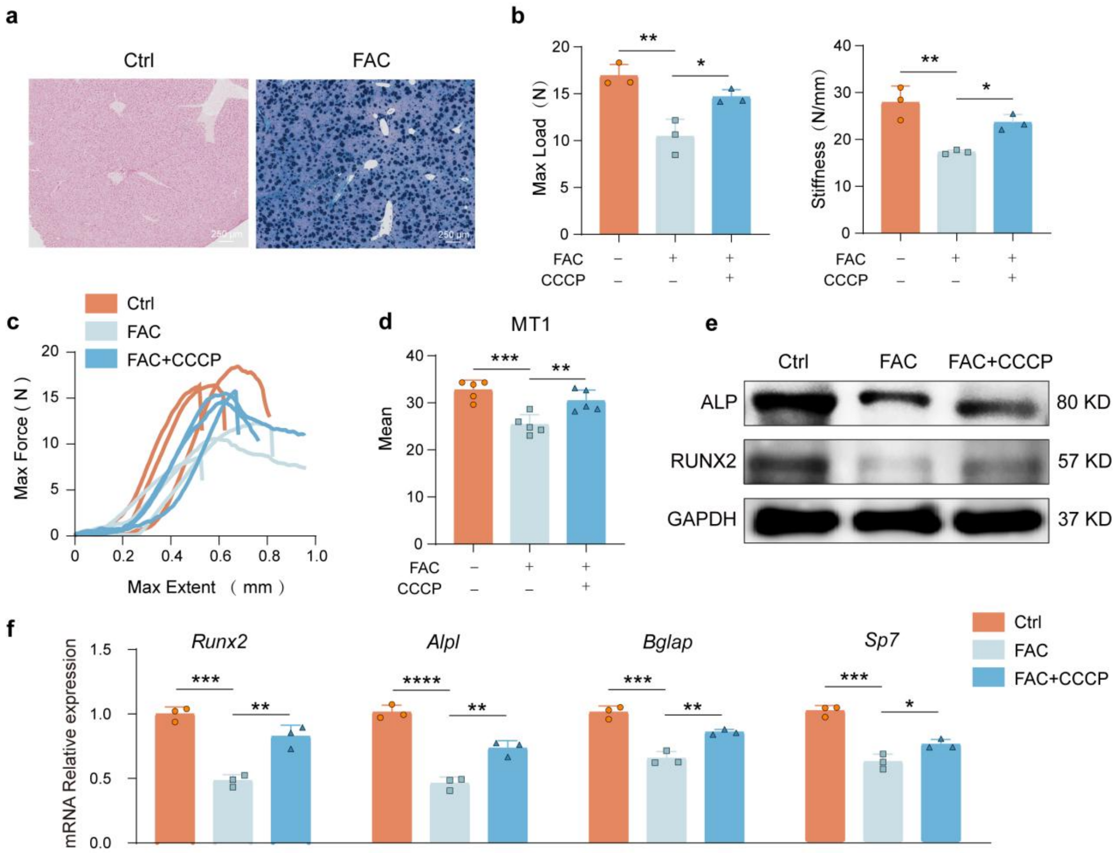

Figure S4. Mitophagy activation restores bone mass in iron-accumulating mice. **(a)** Liver Prussian blue staining for iron deposition in mice treated with or without FAC. Scale bar: 250 μm. **(b-c)** Biomechanical properties assessed by three-point bending test, showing maximum load and stiffness. **(d)** Quantitative analysis of MT-1 immunofluorescence staining. **(e)** Western blot analysis of osteogenic markers (RUNX2, ALP) in BMSCs. **(f)** RT-qPCR analysis of osteogenic genes (*Runx2, Alpl, Bglap, Sp7*) in BMSCs. Data are expressed as mean ± SEM; One-way ANOVA (Tukey's multiple-comparison test); **P* < 0.05; ***P* < 0.01; ****P* < 0.001; *****P* < 0.0001.

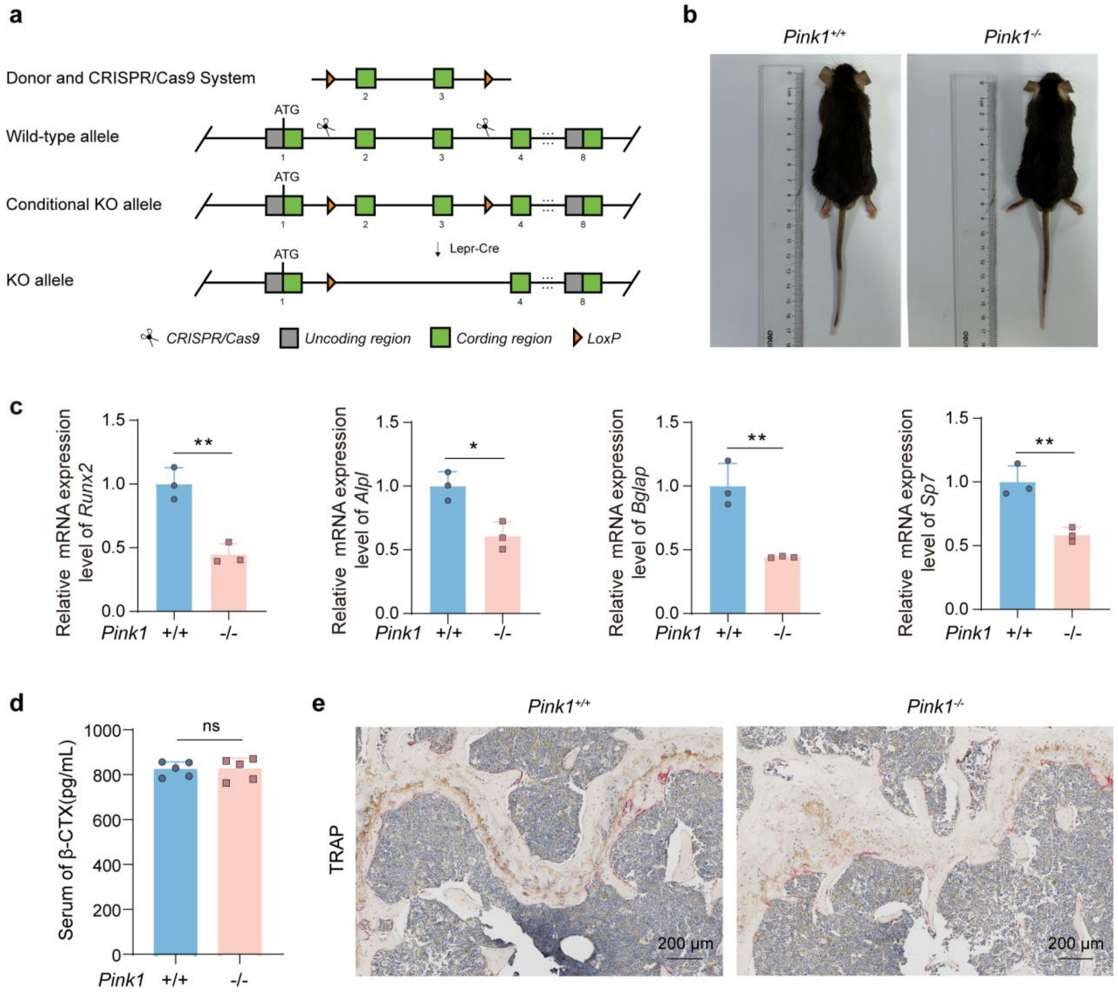

Figure S5. Pink1 deletion in BMSCs induces bone loss independent of iron accumulation. (a) Generation of *Pink1*-floxed and *Pink1*$^{fl/fl}$; Lepr-Cre mice. The schematic diagram illustrates the strategy to delete *Pink1*. LoxP sites are located on exon 2 and exon 3. **(b)** Body length comparison of *Pink1*$^{fl/fl}$ mice and *Pink1*$^{fl/fl}$; Lepr-Cre mice. **(c)** RT-qPCR quantification of osteogenesis marker genes (*Runx2, Alpl, Bglap, and Sp7*) in BMSCs. **(d)** Detection of serum β-CTX levels in *Pink1*$^{fl/fl}$ mice and *Pink1*$^{fl/fl}$; Lepr-Cre mice. **(e)** TRAP staining of tibia paraffin sections from *Pink1*$^{fl/fl}$ mice and *Pink1*$^{fl/fl}$; Lepr-Cre mice. Scale bar: 200 μm. Data are expressed as mean ± SEM; Unpaired 2-tailed Student's *t* test; **P* < 0.05; ***P* < 0.01.

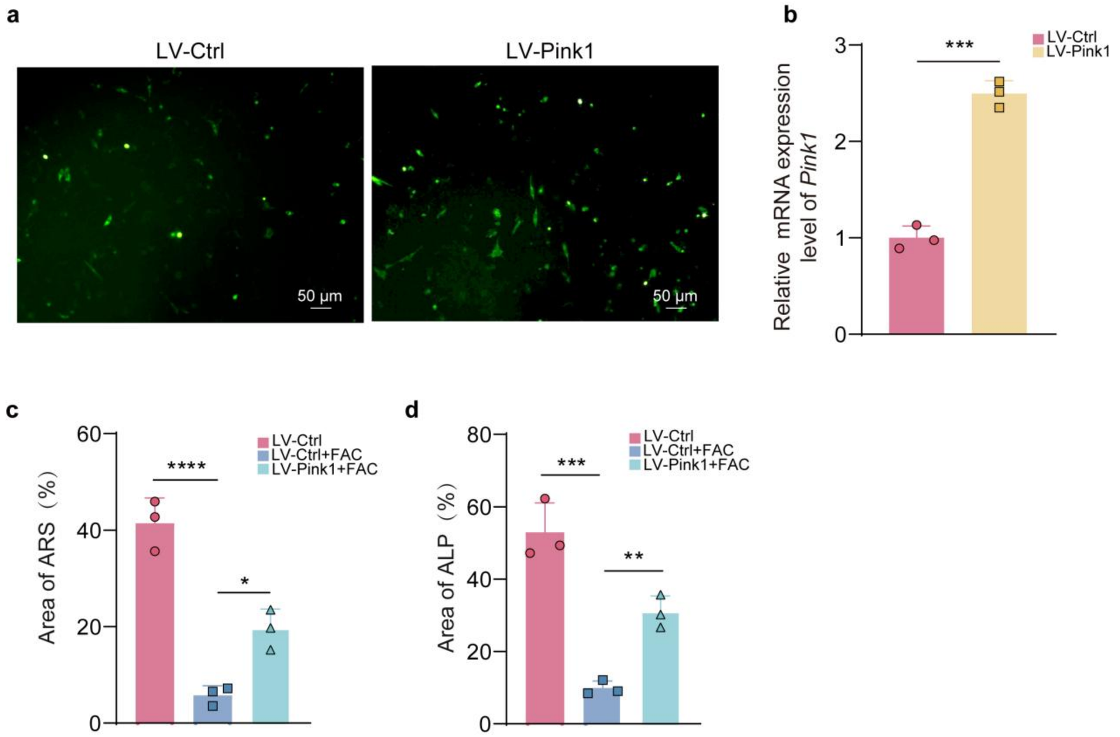

Figure S6. Pink1 overexpression rescues iron accumulation-induced cellular senescence and reverses osteogenic dysfunction in BMSCs. (a) Transfection efficiency of the lentivirus was detected via immunofluorescence staining. Scale bar: 50 μm. **(b)** Detection of *Pink1* overexpression efficiency after lentivirus-mediated *Pink1* overexpression in BMSCs. **(c, d)** Quantification of the area of ARS and ALP staining in the BMSCs from each group (LV-Ctrl group, LV-Ctrl+FAC group, and LV-Pink1+FAC group). Data are expressed as mean ± SEM; One-way ANOVA (Tukey's multiple-comparison test); $*P < 0.05$; $**P < 0.01$; $***P < 0.001$; $****P < 0.0001$.

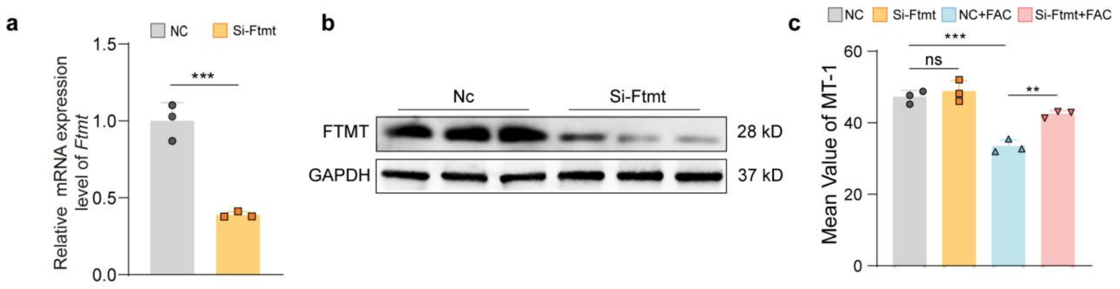

Figure S7. FTMT upregulation during iron accumulation impairs mitophagy by inhibiting PINK1 phosphorylation. (a) Detection of the knockdown efficiency of siRNAs for *Ftmt* knockdown in BMSCs. **(b)** Western blot validation of *Ftmt* knockdown efficiency. **(c)** Quantitative analysis of MT-1 immunofluorescence staining. Data are expressed as mean ± SEM; Unpaired 2-tailed Student's *t* test (a), One-way ANOVA (Tukey's multiple-comparison test) (c); $^{**}P < 0.01$; $^{***}P < 0.001$.

Table S1. Comparison of baseline data in the three groups.

Features	Normal n=3	PMOP n=3	IOP n=3	P1	P2	P3
Age	69.00 ± 7.00	72.00 ± 2.00	72.67 ± 4.16	0.74	0.64	0.98
Ferritin (ng/ml)	117.00 ± 7.00	93.40 ± 28.30	460.00 ± 89.06	0.86	<0.05	<0.05
T-Score	-0.40 ± 0.62	-2.57 ± 0.12	-2.63 ± 0.12	<0.05	<0.05	0.97

Normal: Normal bone mass group; PMOP: Postmenopausal osteoporosis group; IOP: Osteoporosis with iron accumulation. P1: Normal vs PMOP; P2: Normal vs IOP; P3: PMOP vs IOP. $P < 0.05$ was considered statistically significant.
